# Convergent GenX biodegradation by genomically designed and functionally screened synthetic bacterial consortia

**DOI:** 10.3389/fmicb.2026.1848796

**Published:** 2026-06-24

**Authors:** Esaú De la Vega-Camarillo, Jorge Arreola-Vargas, Saurav Kumar Mathur, Rashmi Singla, Smriti Shankar, Cory Klemasevich, Sanjay Antony-Babu, Won Bo Shim

**Affiliations:** 1 Department of Plant Pathology and Microbiology, Texas A&M University, College Station, TX, United States; 2 Institute for Advancing Health Through Agriculture, Texas A&M AgriLife Research, College Station, TX, United States

**Keywords:** bioremediation, enzymatic defluorination, GenX (HFPO-DA), microfluidic assembly, PFAS biodegradation, synthetic microbial consortia

## Abstract

GenX (hexafluoropropylene oxide dimer acid) has been introduced as a replacement for legacy polyfluoroalkyl substances (PFASs) compounds; however, accumulating evidence indicates this C6 ether-PFAS may exhibit comparable toxicity to PFOA while persisting in contaminated water supplies. Biological remediation offers genuine mineralization potential but has been constrained by the exceptional stability of carbon–fluorine bonds. Here, we demonstrate that synthetic bacterial consortia assembled through rational genomic design (GENIA) or microfluidic selection (Community G) achieve rapid GenX biodegradation through metabolic complementarity among functionally specialized taxa. Both consortia removed >80% of GenX within 3 days (GENIA: 94.6%; Community G: 80.6%), with defluorination confirmed by fluoride release (82.6 and 69.6%, respectively). First-order kinetic modeling yielded rate constants of *k* = 0.87 day^−1^ (GENIA) and *k* = 0.64 day^−1^ (Community G), corresponding to half-lives under 1.1 days. Despite pronounced taxonomic divergence, GENIA maintained a stable composition, whereas Community G underwent dramatic restructuring, enriching for specialists of *Salmonella, Pseudomonas*, and *Stenotrophomona*s. Comparative genomic analysis revealed functional equivalence, with no significant differences in PFAS-degrading gene content (*p* = 0.844). Multi-guild community architecture emerged wherein primary degraders (40%−60% abundance) harboring haloacid dehalogenases performed initial C–F bond cleavage, detoxification specialists possessing glutathione S-transferases and fluoride exporters (>90% universal distribution) processed toxic intermediates, and metabolic support taxa enhanced resilience. Molecular docking simulations predicted favorable binding of GenX across phylogenetically diverse dehalogenases (Δ*G* = −7.0 to −8.1 kcal/mol), suggesting thermodynamic compatibility with enzymatic catalysis. These results are predictive and computational; docking cannot confirm enzymatic activity or substrate turnover. Experimental validation (enzyme kinetics, purified protein assays) is required. Critically, GENIA activity did not disrupt indigenous soil microbiomes. The data support that GENIA does not cause detectable diversity loss, a necessary but not sufficient condition for ecological compatibility. These findings establish that functional gene diversity, distributed across metabolically complementary taxa through either rational design or adaptive selection, enables efficient PFAS bioremediation, converting recalcitrant “forever chemicals” into biodegradable substrates through engineered microbial complexity.

## 1 Introduction

Per- and polyfluoroalkyl substances (PFASs) are among the most challenging environmental contaminants of the 21st century. Collectively known as “forever chemicals,” PFAS encompass over 14,000 synthetic compounds characterized by strong carbon–fluorine (C–F) bonds that render them exceptionally resistant to degradation (Evich et al., 2022). This molecular stability, while advantageous for industrial applications, including firefighting foams, nonstick coatings, water-repellent textiles, and food packaging, has led to widespread environmental persistence and bioaccumulation in humans and wildlife (Panieri et al., 2022; Brunn et al., 2023). The U.S. Environmental Protection Agency (EPA) (2022) has identified PFAS contamination at more than 12,000 sites nationwide and drinking water systems serving millions of Americans exceed health advisory levels (Cotruvo et al., 2023). Regulatory pressure on legacy PFAS, particularly perfluorooctanoic acid (PFOA) and perfluorooctane sulfonate (PFOS), has driven industry toward the use of “replacement” compounds marketed as safer alternatives (Cordner et al., 2021). Among these, hexafluoropropylene oxide dimer acid (HFPO-DA), commercially known as GenX, has emerged as the predominant PFOA substitute following its introduction by chemical manufacturers in the 2000s (Guo et al., 2025). GenX is structurally distinct from legacy perfluoroalkyl acids: it features a shorter carbon chain (C6) with an ether linkage, which theoretically reduces its bioaccumulation potential relative to C8 compounds (Brase et al., 2021; Guo et al., 2025). However, this presumed advantage has proven illusory. Environmental monitoring studies have documented widespread GenX contamination in surface waters, groundwater, and drinking water supplies near manufacturing facilities and downstream of industrial discharges (Munoz et al., 2019; Brase et al., 2021). Toxicological studies indicate that GenX exhibits hepatotoxicity, immunotoxicity, and developmental effects comparable to or exceeding those of PFOA in animal models (Conley et al., 2021; Wasel et al., 2023; Wei et al., 2025), prompting the EPA to establish a health advisory level of 10 ng/L for drinking water (U.S. Environmental Protection Agency, 2022). The potential for “regrettable substitution” of legacy PFAS with similarly problematic replacement compounds underscores an urgent need for effective remediation technologies. Current PFAS treatment approaches, including activated carbon adsorption, ion exchange, and advanced oxidation processes, achieve removal but do not mineralize these compounds, merely transferring contamination to waste streams requiring disposal (Verma et al., 2021; Xu et al., 2021; Riegel et al., 2023). Biodegradation, the enzymatic transformation of organic contaminants into less harmful metabolites or complete mineralization, represents an attractive alternative that addresses the root problem rather than relocating it. However, PFAS biodegradation has historically been considered impractical due to the exceptional stability of C–F bonds (bond dissociation energy: 485 kJ/mol), which few biological systems can cleave (Grgas et al., 2023; Berhanu et al., 2023; Wackett, 2025). Recent breakthroughs have challenged this paradigm. Studies employing enrichment cultures from contaminated environments have demonstrated measurable biodegradation of PFOS and PFOA, with removal efficiencies ranging from 46 to 69% for PFOS and 16%−60% for PFOA under specific incubation periods and optimized environmental conditions (Huang and Jaffé, 2019; Leung et al., 2022; Liang and Ma, 2024; Lorah et al., 2024; Sorn et al., 2025; Yi et al., 2016). Genomic analyses of these degraders have identified putative defluorinase and reductive dehalogenase genes potentially responsible for C–F bond cleavage, although direct enzyme characterization remains limited (Harris et al., 2022; Chetverikov et al., 2024; Farajollahi et al., 2024; Berhanu et al., 2023). Crucially, these studies have primarily focused on legacy perfluoroalkyl acids; biodegradation of replacement ether-PFAS like GenX remains largely uncharacterized (Leung et al., 2022; Kumar et al., 2023; Mantripragada et al., 2023). The structural differences between GenX (ether linkage, carboxylate head group) and legacy PFAS (perfluoroalkyl chain, sulfonate/carboxylate termini) raise fundamental questions about substrate promiscuity: can microbial communities evolved for PFOA/PFOS degradation also transform GenX? (Jin et al., 2023) Do ether bonds present different enzymatic challenges than perfluorinated chains? What genes and pathways are required for GenX biotransformation? (Hu and Scott, 2024; Skinner et al., 2025; Sorn et al., 2025). Our laboratory has previously developed synthetic microbial consortia capable of degrading legacy PFAS through complementary approaches. Using a modified Prospector microfluidic platform with controlled cell loading densities, we systematically assembled Communities A–H from agricultural soil microbiomes, achieving up to 91.5% PFOS removal in bioreactor-scale experiments (De la Vega-Camarillo et al., 2026). Random forest classification identified substrate-specific keystone taxa, *Atlantibacter* and *Stenotrophomonas* for PFOS, *Enterobacter* for PFOA, indicating distinct enzymatic pathways for different PFAS structures. In parallel, we constructed GENIA (Genomically and Environmentally Networked Intelligent Assemblies), a rationally designed consortium in which strains were selected based on the genomic presence of predicted defluorination genes rather than empirical screening. GENIA demonstrated superior degradation of recalcitrant PFOA (78.93%) compared with microfluidically assembled communities (typically < 25%), highlighting the advantage of genomic-informed design for capturing rare yet essential catabolic genes (De la Vega-Camarillo et al., 2026). However, the performance of these established consortia against GenX, a structurally distinct ether-PFAS, remained unknown. This knowledge gap is critical for two reasons. First, from a fundamental perspective, determining whether PFAS-degrading consortia exhibit substrate promiscuity across structural classes (perfluoroalkyl vs. ether-PFAS) informs our understanding of enzyme specificity and the evolutionary potential to adapt to emerging contaminants. Second, from an applied perspective, demonstrating broad-spectrum PFAS-degradation capacity is essential for real-world remediation scenarios involving contaminated sites that contain complex mixtures of legacy and replacement compounds. If consortia optimized for PFOA/PFOS cannot degrade GenX, separate treatment systems would be required, thereby dramatically increasing remediation complexity and cost. In this study, we comprehensively characterized GenX biodegradation by nine synthetic microbial consortia, integrating experimental validation with genomic and computational approaches to elucidate mechanistic pathways. We first evaluated GenX removal efficiency across all consortia using LC-MS/MS quantification and compared performance against legacy PFAS (PFOS, PFOA) to establish structure-activity relationships. We validated genuine defluorination, rather than abiotic disappearance via adsorption or volatilization, using fluoride liberation assays that quantified the release of free fluoride ions. To investigate the genetic basis of degradation capacity, we performed comparative genomic analysis identifying putative defluorinase genes and assessed their distribution across high- and low-performing consortia. Molecular docking simulations predicted GenX-enzyme binding energies and active site interactions, while molecular dynamics modeling proposed theoretical degradation pathways and energy barriers for C–F bond cleavage. Finally, temporal microcosm experiments established first-order kinetic parameters, enabling predictive modeling for bioreactor-scale-up. Our results demonstrate that established PFAS-degrading consortia maintain excellent efficacy against GenX (>91% removal by all consortia, 100% by GENIA), with ether-PFAS exhibiting substantially higher biodegradability than legacy perfluoroalkyl compounds. Genomic and computational analyses reveal that this performance derives from substrate-compatible defluorinase enzymes that recognize both ether- and alkyl-PFAS structures, albeit with varying efficiency. These findings establish both the fundamental enzymatic basis and practical feasibility of biological treatment for GenX-contaminated sites, providing candidate consortia and preliminary kinetic parameters that support further development toward field application, pending scale-up validation.

## 2 Materials and methods

### 2.1 Bacterial consortia

Nine synthetic microbial consortia were evaluated for their capacity to biodegrade GenX in this study. Eight consortia (Communities A–H) and GENIA (Genomically and Environmentally Networked Intelligent Assemblies) were previously assembled and characterized as described in De la Vega-Camarillo et al. (2026). Briefly, Communities A–H were assembled via high-throughput microfluidic functional screening from agricultural soil microbiomes (De la Vega-Camarillo et al., 2026). At the same time, GENIA was rationally designed through genomic-informed selection based on predicted defluorination gene content. All consortia were maintained as glycerol stocks (20% v/v) at −80 °C. For experimental use, consortia were cultured in M9 minimal medium (6.78 g/L Na_2_HPO_4_, 3 g/L KH_2_PO_4_, 0.5 g/L NaCl, 1 g/L NH_4_Cl, 2 mM MgSO_4_ 0.1 mM CaCl_2_. Standardized suspensions were prepared by adjusting all consortia to the same cell density (OD_600_ = 0.05) after centrifugation (5,000 × g, 10 min, 4 °C), washing twice with sterile phosphate-buffered saline (PBS, pH 7.4), and resuspension in fresh M9 medium.

### 2.2 Chemicals and reagents

GenX (hexafluoropropylene oxide dimer acid ammonium salt, HFPO-DA, CAS 13252-13-6, >95% purity) was obtained from US Biological Life Sciences (Salem, MA, USA). Stock solutions (10,000 ng/ml) were prepared in ultrapure water (Milli-Q, 18.2 MΩ·cm) and stored at 4 °C in amber glass vials with PTFE-lined caps to minimize volatilization and photodegradation. Working solutions were freshly prepared for each experiment. All organic solvents (acetonitrile, methanol, formic acid; HPLC grade) were purchased from Fisher Scientific (Pittsburgh, PA, USA). SPADNS (4,5-dihydroxy-3-[(4-sulfophenyl)azo]-2,7-naphthalenedisulfonic acid trisodium salt) reagent for fluoride determination was obtained from Hach Company (Loveland, CO, USA).

### 2.3 Microplate screening assays

Initial metabolic viability and GenX degradation screening were conducted in 96-well microplates (Corning, flat-bottom, black walls, clear bottom). Each well contained 190 μl of M9 minimal medium supplemented with 500 ng/ml GenX and was inoculated with 10 μl of a standardized consortium suspension (all consortia adjusted to an identical cell density of 10^5^ cells/ml, yielding a final inoculum density of ~5 × 10^3^ cells/well in a total volume of 200 μl). Resazurin (final concentration 0.001% w/v) was added as a redox indicator to assess metabolic activity. For all microplate-related work, experiments were conducted using an Opentrons-OT2 to ensure reproducibility and consistency throughout the project. Microplates were incubated at 28 °C with orbital shaking (150 rpm) in the dark to prevent photodegradation. Measurements were performed at 72 h post-inoculation using a SpectraMax M5 microplate reader (Molecular Devices, San Jose, CA, USA). Growth was quantified by optical density at 600 nm (OD_600_), with ΔOD_600_ calculated as OD_600, 72h_ – OD_600, 0h_. Metabolic activity was assessed by measuring fluorescence intensity (excitation 560 nm, emission 590 nm) during resazurin reduction. Background fluorescence from media-only wells was subtracted, and resazurin reduction fluorescence (RFU) fold-change was calculated as (RFU_72h_ – RFU_0h_)/RFU_0h_ (Balbaied and Moore, 2020). For GenX degradation analysis, supernatants were collected after centrifugation (3,000 × g for 10 min) and analyzed by LC-MS/MS as described below. Aliquots of supernatants were reserved for fluoride quantification via the SPADNS micromethod. All microplate assays were performed in quadruplicate with appropriate controls: (1) uninoculated media with GenX (abiotic control, to quantify abiotic losses); (2) heat-killed consortia (autoclaved 121 °C, 30 min; sterility confirmed by absence of growth on R2A agar after 72 h incubation at 28 °C) with GenX (adsorption/abiotic control); and (3) active consortia without GenX (baseline metabolic activity). Here, we note that autoclaving may alter soil physicochemical properties (pH, soluble carbon, metal ions), which could affect GenX sorption differently from active treatments; this is discussed as a limitation.

### 2.4 Microcosm biodegradation experiments

Temporal GenX biodegradation was evaluated in soil microcosms to assess performance under environmentally relevant conditions. Microcosms were established in sterile culture jars (CultureJar^TM^ G9 220 ml) containing 80 g of soil substrate (Meng et al., 2023). Two soil substrate types were prepared: (1) sterile control substrate consisting of autoclaved field soil:sand mixture (3:1 w/w) subjected to three consecutive autoclaving cycles (121 °C, 60 min each, with 24 h intervals between cycles) to ensure complete elimination of indigenous microbiota (enabling unambiguous tracking of GENIA performance without background microbial signals; heat-killed GENIA controls were confirmed sterile by R2A agar plating (no growth after 72 h at 28 °C), and (2) inoculated treatment consisting of the sterile soil:sand mixture inoculated with soil tea to establish an indigenous microbiome, enabling ecological impact assessment, prepared from environmental samples. Fresh soil samples were collected from the cotton rhizosphere at Texas A&M Brazos Bottom Experimental Farm Station (30.551157826632647, −96.43135829477686), TX, USA, in April 2025. Samples were collected from 0 to 15 cm depth using sterile technique and transported on ice to the laboratory within 2 h of collection. Soil tea was prepared by suspending 400 g fresh rhizosphere soil in 1,000 ml; sterile phosphate buffer (50 mM, pH 7.2) and stirring for 1 h (200 rpm, room temperature). Subsequently, 860 ml of sterile 50% glycerol was added (final glycerol 23% v/v for cryopreservation at −80 °C). The suspension was allowed to settle for 10 min, and the supernatant was collected and stored at −80 °C in conical 50 ml tubes. This soil tea (5 ml per jar) was used to inoculate the sterile soil:sand substrate. Residual glycerol per jar was estimated at < 0.1% w/w in the soil matrix; applied uniformly across all treatment groups, including controls, this concentration is not expected to selectively confound GenX degradation results, though it is acknowledged as a minor carbon source. Soil moisture was adjusted to 60% water-holding capacity using sterile deionized water. GenX was added to achieve a final concentration of 2,000 ng/ml in soil pore water (calculated from measured water content). After thorough mixing, 1 ml standardized consortium suspension was spot-inoculated onto the soil surface. Microcosms were incubated at 28 °C in the dark with daily moisture adjustment; gravimetric moisture content was verified at each sampling time point by weighing jars before and after moisture addition to confirm pore water volume remained within ±5% of the target value throughout the experiment. Sampling was performed at 0, 1, 2, and 3 days post-inoculation (*n* = 4 replicate jars per timepoint per treatment). At each time point, the entire jar contents were homogenized. Subsamples were collected for: (1) GenX quantification via single solvent extraction of 0.5 g soil in 4.5 ml methanol followed by centrifugation and LC-MS/MS analysis of the supernatant, recovery efficiency was not independently validated; relative comparisons across treatment groups remain valid under uniform recovery assumptions; (2) fluoride quantification via aqueous extraction followed by the SPADNS method; and (3) DNA extraction for microbial community composition analysis.

### 2.5 Fluoride quantification

Fluoride liberation was quantified using the SPADNS (4,5-dihydroxy-3-[(4-sulfophenyl)azo]-2,7-naphthalenedisulfonic acid) colorimetric micromethod adapted for 96-well microplates. This method is based on the bleaching of the red SPADNS-zirconium complex by fluoride ions, with absorbance decrease proportional to fluoride concentration (Fernando et al., 2023). SPADNS reagent was prepared according to EPA Method 340.1 with modifications for microplate format. For microplate supernatants, 50 μl supernatant was mixed with 50 μl SPADNS reagent in microplate wells. For microcosm extracts, 0.5 g of sample was first diluted 1:10 in deionized water to partially reduce matrix interference, then mixed with SPADNS reagent at the same dilution factor. We acknowledge that SPADNS is susceptible to interference from soil ions (Al^3+^, Fe^3+^, Cl^−^), therefore matrix-matched calibration curves were used to partially compensate. Matrix-matched calibration has been validated against ISE measurements. Absorbance was measured at 570 nm using the SpectraMax M5 microplate reader after 5 min reaction time at room temperature. Calibration curves (0.1–10 mg/L F^−^) were generated using certified NaF standards (Fisher Scientific) prepared in the same matrix as samples. Standard curves were linear (*R*^2^ > 0.998), and separate matrix-matched standards were used for M9 minimal medium (microplate experiments) and methanol soil extracts (microcosm experiments), controlling for matrix-specific ionization effects. Method detection limit was 0.05 mg/L F^−^. Defluorination efficiency was calculated as: Defluorination (%) = ([F^−^] measured/[F^−^] theoretical maximum) × 100, where theoretical maximum fluoride was calculated based on GenX molecular weight (330.05 g/mol) and fluorine content (11 atoms per molecule, 63.3% by mass).

### 2.6 LC-MS/MS quantification of GenX

GenX concentrations were quantified using liquid chromatography-tandem mass spectrometry. The target compound was detected and quantified on a triple quadrupole mass spectrometer (Altis, Thermo Scientific, Waltham, MA, USA) coupled to a binary pump HPLC system (Vanquish, Thermo Scientific). MS parameters were optimized for GenX under direct infusion at 5 μl/min to identify the selected reaction monitoring (SRM) transitions (precursor/product fragment ion pair) with the highest intensity. Samples were maintained at 4 °C in the autosampler before injection. The injection volume was 10 μl. Chromatographic separation was achieved on a Hypersil Gold column (5 μm particle size, 50 × 3 mm i.d., Thermo Scientific, Waltham, MA, USA) at 30 °C using a 9.5-min solvent gradient. Mobile phases consisted of solvent A (water with 0.1% formic acid v/v) and solvent B (acetonitrile with 0.1% formic acid v/v) at a flow rate of 0.6 ml/min. The gradient program was as follows: 0–1.0 min, 5% B; 1.0–6.0 min, 5%−95% B linear gradient; 6.0–8.0 min, 95% B hold; 8.0–8.5 min, 95%−5% B; 8.5–9.5 min, 5% B re-equilibration. Mass spectrometry was performed in negative electrospray ionization (ESI-) mode. The spray voltage was 2,500 V, sheath gas pressure 50 arbitrary units, auxiliary gas pressure 10 arbitrary units, ion transfer tube temperature 350 °C, and vaporizer temperature 300 °C. The precursor ion [M–H]^−^ at m/z 285.0 was fragmented to product ions at m/z 169.0 (quantifier, collision energy 15 eV) and m/z 119.0 (qualifier, collision energy 25 eV). Retention time for GenX was 4.8 ± 0.2 min with a dwell time of 50 ms per transition. Sample acquisition and analysis were performed using TraceFinder 3.3 software (Thermo Scientific). Calibration curves (1–5,000 ng/ml) were generated using matrix-matched standards. The method quantification limit was 1.5 ng/ml. Quality control samples were analyzed every 10 samples with acceptance criteria of ±15% of nominal concentration.

### 2.7 DNA extraction

Total community DNA was extracted from microcosm soil samples (0.5 g) using the ZymoBIOMICS DNA Miniprep Kit (Zymo Research, Irvine, CA, USA) according to the manufacturer's protocol, with bead-beating to enhance cell lysis. DNA concentration was quantified using a Qubit 4.0 fluorometer (Invitrogen, Carlsbad, CA, USA) with the dsDNA High Sensitivity Assay Kit. DNA quality was assessed via spectrophotometry (NanoDrop One, Thermo Scientific) with A_260_/A_280_ ratios of 1.8–2.0 considered acceptable. For whole-genome sequencing, pure culture isolates were grown to mid-log phase in R2A broth (28 °C, 150 rpm, 24 h). Cells were harvested by centrifugation (5,000 × g, 10 min, 4 °C), and high-molecular-weight genomic DNA was extracted using the ZymoBIOMICS DNA Miniprep Kit (Zymo Research, Irvine, CA, USA) according to the manufacturer's instructions. DNA integrity was confirmed by agarose gel electrophoresis (1.0% w/v), with intact high-molecular-weight bands (>20 kb) required for downstream sequencing.

### 2.8 Sequencing

Both full-length 16S rRNA gene sequencing (community samples) and whole-genome sequencing (isolates) were performed by Plasmidsaurus (Eugene, OR, USA) using Oxford Nanopore Technologies PromethION platform with R10.4.1 flow cells. For 16S rRNA gene sequencing, full-length 16S rRNA genes were amplified and sequenced using the latest v14 library-prep chemistry, with barcoded full-length amplification using Plasmidsaurus in-house sequencing primers. Libraries were sequenced with a primer-free protocol on R10.4.1 flow cells, producing full-length sequencing reads for each amplicon. Basecalling was performed using the Super-Accurate model, with quality filtering to retain reads with a *Q*-score ≥ 10 and a length of 400–3,000 bp. Taxonomic classification employed emu v3.5.1 against rrnDB v5.6 and the NCBI Targeted Loci databases to identify bacterial, archaeal, and eukaryotic species. Raw data were delivered in compressed fastq format (.fastq.gz) (Curry et al., 2022). For whole-genome sequencing, amplification-free long-read sequencing libraries were constructed using the latest v14 library-preparation chemistry, including minimal fragmentation of input genomic DNA via tagmentation in a sequence-independent manner. Libraries were sequenced with a primer-free protocol using R10.4.1 flow cells. Basecalling employed Dorado Super-Accurate basecalling with default Q10 quality filtering. Quality filtering was performed with Filtlong v0.2.1 to remove 5% of the lowest-quality reads using the default parameters. Genome size was estimated using Autocycler helper functions, and multiple subsampled read sets were generated using Autocycler with optimal coverage for each subsample. Multiple assemblies were performed using Autocycler with three different assemblers: Flye v2.9.6+ with parameters optimized for high-quality ONT reads, Hifiasm for generating high-quality assemblies from long reads, and Plassembler v1.8.0+ for detecting and assembling plasmids. Low-depth and small contigs were removed, and assemblies were compressed, clustered, trimmed, resolved, and cleaned using Autocycler to identify the best consensus assembly from all assemblers (Wick et al., 2025). The assembly was rotated to start at the optimal position using dnaapler. The Flye assembly was performed using Medaka v1.8.0 on the filtered reads. Assembly quality was assessed through genome completeness and contamination analysis with CheckM v1.2.2 (Parks et al., 2015). Functional annotation was performed with Bakta v1.11 (Schwengers, 2025). Contig analysis was conducted with Bandage v0.8.1. Species and plasmid identification employed Mash v2.3 against RefSeq genomes and plasmids, and Sourmash v4.6.1 against GenBank. Assembled genome accuracy is typically Q50–60, corresponding to 99.999%−99.9999% accuracy (one error per 100,000–1,000,000 bases).

### 2.9 Bioinformatics analysis

Taxonomic abundance data from emu classification were imported into Python 3.9 for downstream analysis. Alpha diversity metrics (Shannon index, Simpson index, observed species richness) were calculated using the scikit-bio library v0.5.9 (Aton et al., 2025). Beta diversity was assessed using Bray–Curtis dissimilarity matrices and visualized via principal coordinates analysis (PCoA) with SciPy v1.11.3 and Matplotlib v3.8.0 (The Matplotlib Development Team, 2025). Differential abundance analysis between treatments and time points was performed using the statsmodels library (version 0.14.0) with negative binomial generalized linear models and false discovery rate correction via the Benjamini–Hochberg method. Significant taxa were defined as those with adjusted *p* < 0.05 and log_2_ fold-change >1.5. Annotated genomes generated with Bakta were analyzed for functional gene content using custom Python scripts. Genes potentially involved in defluorination and related xenobiotic transformation processes were identified through sequence similarity searches using BLAST+ v2.14.0 against custom reference databases composed of experimentally characterized and literature-reported sequences of dehalogenases, fluoroacetate dehalogenases, and organophosphate hydrolases retrieved from GenBank and UniProt (Cabrera et al., 2022). Searches were conducted using an e-value threshold of < 10^−5^ and a minimum amino acid identity of 40%. This threshold captures divergent dehalogenase homologs, consistent with environmental genomics practice; we acknowledge it may include remote homologs with uncertain function. Hits were additionally filtered by query coverage (≥50%) and inspection of conserved catalytic residues. *A* ≥ 60% identity threshold is recommended when specificity is prioritized. Biosynthetic gene clusters were predicted with antiSMASH v8.0 (Blin et al., 2025), and metabolic pathway reconstruction and functional annotation were performed via KEGG Orthology mapping with BlastKOALA (Kanehisa et al., 2016). Comparative genomic analyses were performed using OrthoFinder v3.0 to identify orthologous gene groups across sequenced isolates (Emms et al., 2025). Genome quality metrics, including completeness, contamination, and strain heterogeneity, were assessed using CheckM. Only genomes exhibiting >95% completeness and < 5% contamination were retained for downstream analyses.

### 2.10 Statistical analysis

All statistical analyses were performed in Python 3.9. Differences in alpha diversity (Shannon index) among treatment groups were assessed using Kruskal–Wallis tests with Dunn *post-hoc* tests and Benjamini–Hochberg FDR correction (α = 0.05; *n* = 4 biological replicates per group). Beta diversity differences were evaluated using PERMANOVA (999 permutations) on Bray–Curtis dissimilarity matrices. GenX degradation and defluorination efficiencies between active and inactive consortia were compared using independent two-sample Welch *t*-tests with Benjamini–Hochberg correction. Differences in PFAS-related gene content between consortia were assessed using the Wilcoxon rank-sum test. First-order kinetic models were fit by nonlinear least-squares regression and validated using *R*^2^ (>0.99 for both consortia). Spearman correlations were used for co-occurrence network edges (threshold |ρ| >= 0.55) and taxon-degradation associations. Data are presented as mean ± SD. Significance thresholds: ^*^p < 0.05, ^**^p < 0.01, ^***^p < 0.001; ns = not significant (*p* > 0.05).

## 3 Results

**Figure 1:**
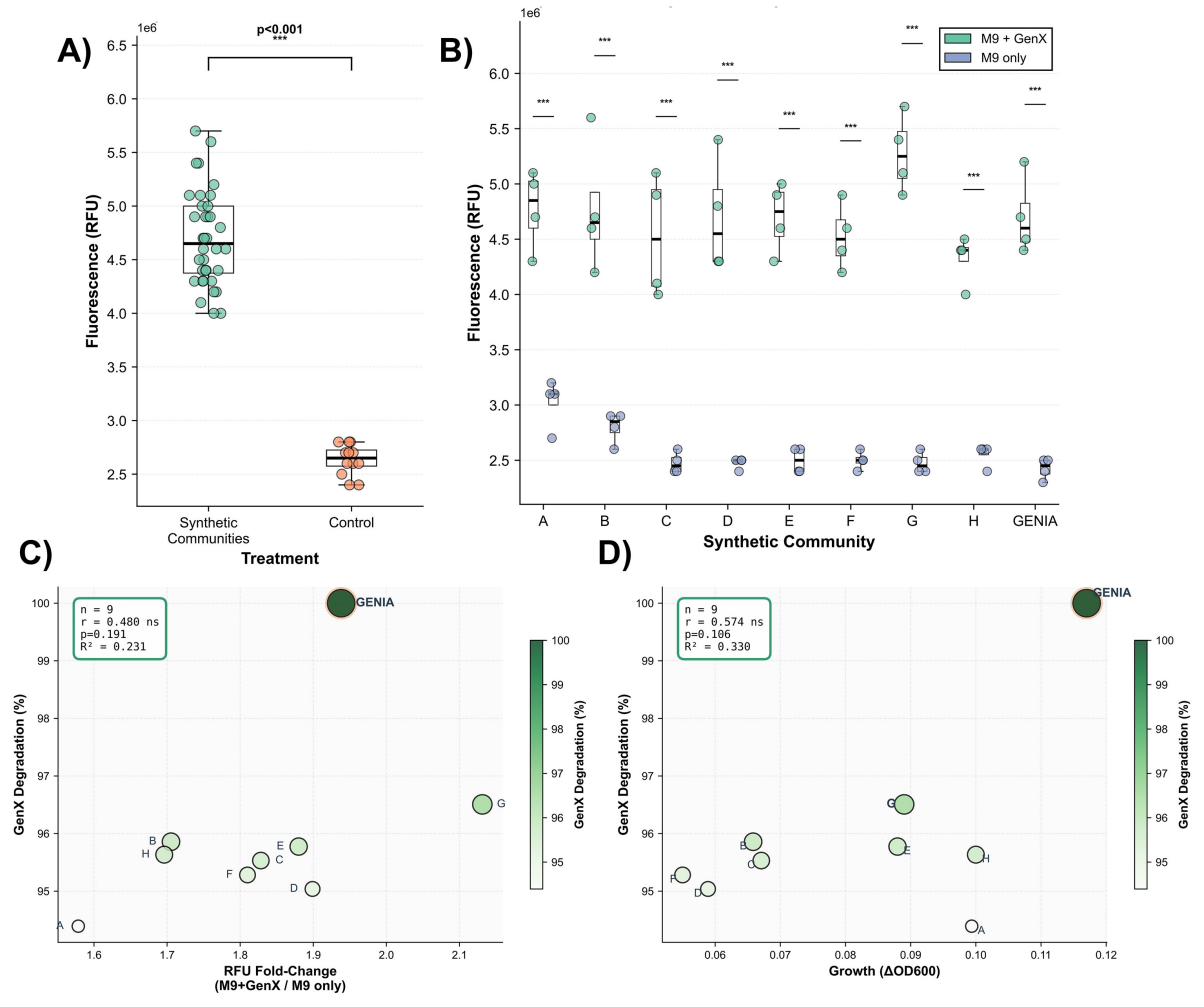
Performance screening of synthetic microbial consortia for GenX biodegradation. **(A)** Overall metabolic activity of nine synthetic communities vs. sterile controls. Resazurin reduction fluorescence (RFU) was significantly higher in all synthetic communities exposed to GenX (Synthetic Communities, teal; ~4.0–5.7 × 10^6^ RFU) compared to uninoculated controls (Control, orange; ~2.5–2.8 × 10^6^ RFU; *p* < 0.001, Welch *t*-test). Box plots show median (center line), interquartile range (box), and min–max range (whiskers); individual points represent biological replicates (*n* = 4 per consortium). **(B)** Community-specific metabolic responses. Fluorescence in M9 minimal medium supplemented with GenX (M9 + GenX, teal boxes) vs. M9-only controls (M9 only, purple boxes) for each consortium (A–H and GENIA). All communities exhibited significantly elevated metabolic activity in the presence of GenX (***p < 0.001). GENIA and Community G achieved the highest fluorescence values. Asterisks denote significance levels from pairwise comparisons (***p < 0.001). **(C)** Relationship between metabolic activity (RFU fold-change, M9 + GenX normalized to M9 only) and GenX degradation efficiency (%). Circle size is proportional to ΔOD_600_ (biomass accumulation). GENIA achieved the highest degradation and RFU fold-change (>96%, 2.1 × ). Pearson correlation: *r* = 0.480, *p* = 0.191, *R*^2^ = 0.231 (*n* = 9; not significant). **(D)** Relationship between growth (ΔOD_600_) and GenX degradation efficiency (%). GENIA achieved the highest values in both metrics. Pearson correlation: *r* = 0.574, *p* = 0.106, *R*^2^ = 0.330 (*n* = 9; not significant), indicating a moderate positive trend between biomass accumulation and degradation capacity. Circle color gradient (light to dark green) represents GenX degradation efficiency (%).

To evaluate the GenX biodegradation capacity of established PFAS-degrading consortia, we first assessed metabolic activity and degradation performance across nine synthetic communities (Communities A–H and GENIA) in microplate screening assays. Metabolic activity, measured as relative fluorescence units (RFU), was significantly higher in all synthetic communities in media containing GenX (4.5–5.7 RFU) compared to sterile controls (2.6–2.8 RFU, *p* < 0.001; Figure 1A), observation suggests active cellular metabolism in the presence of GenX, taking into account that fluorescence reflects general reductive metabolic activity and should not be interpreted as evidence that GenX serves as the primary carbon source; M9 medium components may also support background metabolism.

Individual community analysis revealed differential performance patterns (Figure 1B). Under M9 minimal medium supplemented with GenX (M9 + GenX), all communities exhibited significantly higher metabolic activity than M9-only controls (*p* < 0.001), indicating that GenX serves as a bioavailable carbon source that sustains community metabolism. Communities B, C, D, E, and F demonstrated intermediate performance (4.6–5.1 RFU), while Communities A, G, and H achieved slightly higher values (5.0–5.7 RFU). Notably, GENIA and Community G emerged as top performers, with metabolic activity levels of 5.3 ± 0.3 RFU and 5.7 ± 0.2 RFU, respectively, significantly exceeding those of other consortia and validating their selection for downstream characterization.

### 3.1 Metabolic activity correlates moderately with GenX degradation efficiency

To assess whether metabolic activity and biomass production predict degradation performance, we quantified relationships between RFU fold-change, ΔOD_600_, and GenX removal across all nine communities (Figures 1C, D). GenX degradation percentages ranged from 94.4% (Community A) to 100% (GENIA), with most communities achieving >95% removal, demonstrating consistently high performance across diverse taxonomic configurations.

Analysis of RFU fold-change (normalized to M9-only controls) revealed that Community G and GENIA achieved the highest metabolic enhancements (2.13 × and 1.94 × , respectively), significantly exceeding other consortia (1.58 × -1.90 × ). However, correlation analysis between RFU fold-change and GenX degradation showed only moderate association (Pearson *r* = 0.480, *p* = 0.191, *R*^2^ = 0.231, *n* = 9), while ΔOD_600_ demonstrated slightly stronger correlation with degradation efficiency (Pearson *r* = 0.574, *p* = 0.106, *R*^2^ = 0.330, *n* = 9). Neither correlation was statistically significant, indicating that while biomass accumulation is a more reliable predictor than metabolic activity alone, degradation capacity is not determined by either metric in isolation.

Despite these nonsignificant correlations, both GENIA and Community G demonstrated consistent coupling between high metabolic activity, substantial biomass production (ΔOD_600_ = 0.117 and 0.089, respectively), and near-complete GenX removal, confirming robust community function and justifying their selection for further mechanistic investigation.

### 3.2 Community G exhibits dynamic taxonomic restructuring during GenX degradation

**Figure 2:**
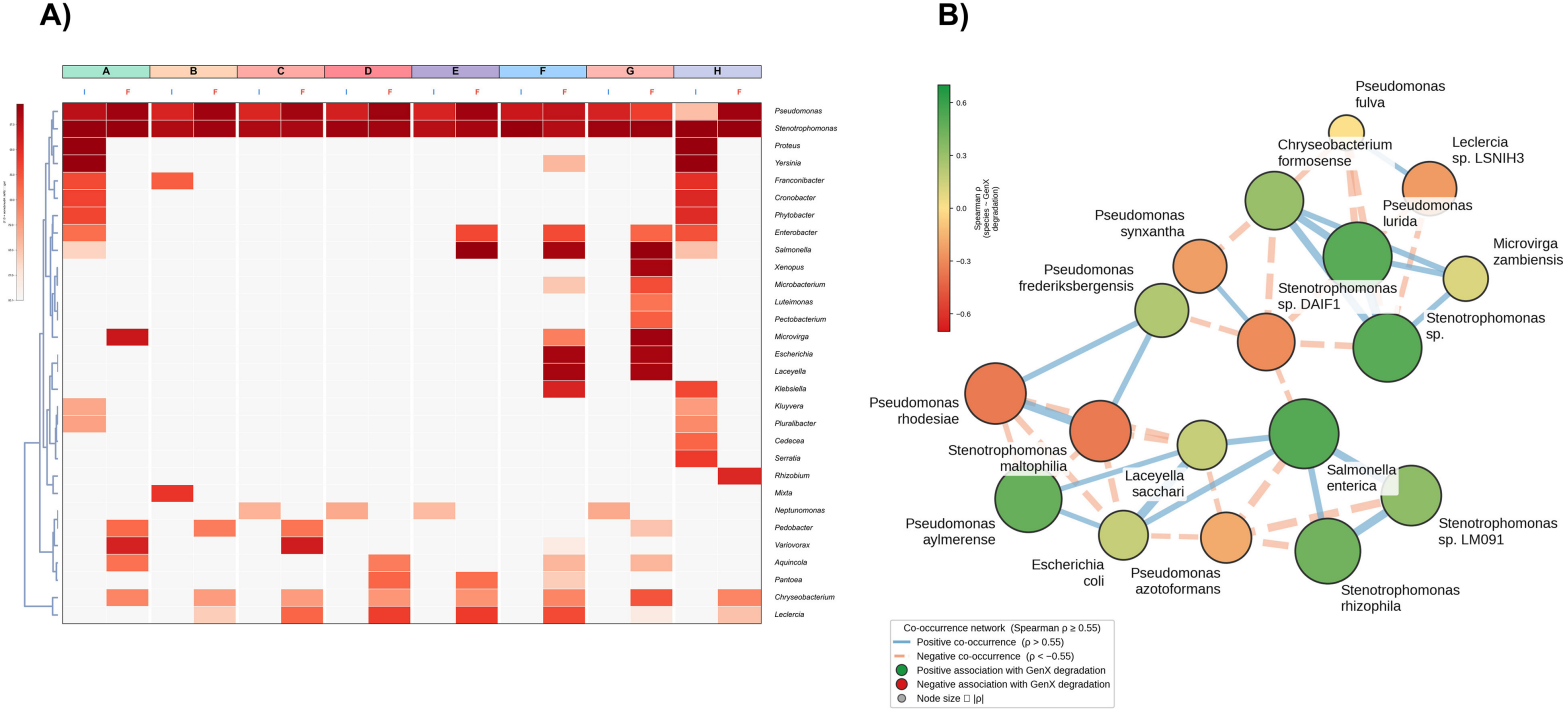
Taxonomic composition and co-occurrence network of synthetic communities during GenX exposure. **(A)** Heatmap of bacterial genus-level relative abundance across Communities A–H at initial (I) and final (F) time points, as determined by full-length 16S rRNA gene sequencing (Oxford Nanopore Technology). Color intensity represents relative abundance (dark red = high, white = absent). A hierarchical dendrogram (left) shows clustering of taxa by abundance patterns across communities. Community G exhibits the most pronounced compositional shifts between initial and final time points, with strong enrichment of *Salmonella, Pseudomonas*, and *Stenotrophomonas* spp. **(B)** Co-occurrence network of bacterial taxa in Community G during GenX biodegradation. Edges represent significant Spearman correlations (|ρ| ≥ 0.55) between taxa: solid blue lines = positive co-occurrence (ρ > 0.55); dashed salmon lines = negative co-occurrence (ρ < −0.55). Node color indicates association with GenX degradation efficiency: green = positive association, orange/red = negative association. Node size is proportional to |ρ| with GenX degradation. Color scale bar (right) shows Spearman ρ values from −0.6 (negative) to +0.6 (positive).

High-throughput full-length 16S rRNA gene sequencing revealed distinct taxonomic profiles across the eight synthetic communities and highlighted Community G's unique compositional dynamics (Figure 2A). The heatmap analysis demonstrated that *Pseudomonas* and *Stenotrophomonas* dominated across treatments A–H, with relative abundances varying temporally and between communities. Community G was characterized by substantial taxonomic shifts from the initial to the final time point, indicating dynamic community restructuring in response to GenX exposure.

Specifically, Community G exhibited dramatic enrichment of key taxa during active degradation: *Salmonella enterica* (0%−22.3%, Δ+22.3%), *Pseudomonas tolaasii* (0.1%−22.3%, Δ+22.2%), *Stenotrophomonas* sp. LM091 (0%−17.3%, Δ+17.3%), and *Stenotrophomonas* sp. MYb57 (0%−16.6%, Δ+16.6%). This compositional plasticity suggests that Community G underwent selective enrichment for PFAS-degrading taxa, in contrast to the more stable compositions observed in other consortia (Figure 2B). These pronounced taxonomic shifts, coupled with high degradation performance, positioned Community G as an ideal system for investigating the relationship between community dynamics and functional capacity.

### 3.3 GENIA and community G exhibit comparable broad-spectrum PFAS degradation capacity

To evaluate substrate specificity across structurally distinct PFAS compounds, we tested GENIA and Community G against GenX (C6 ether-PFAS), PFOS (C8 perfluoroalkyl sulfonate), and PFOA (C8 perfluoroalkyl carboxylate) in parallel degradation assays (Supplementary Figure S1). Both consortia achieved excellent GenX removal (GENIA: 100%; Community G: 96.5%), significantly outperforming their activity against legacy PFAS compounds (*p* < 0.001).

For PFOS, both communities demonstrated substantial but reduced degradation relative to GenX: GENIA achieved 93.7 ± 0.3% removal, while Community G achieved 86.8 ± 1.0% removal, with a significant difference between consortia (*p* = 0.001). PFOA proved more recalcitrant, with GENIA removing 78.9 ± 1.3% and Community G removing 22.6 ± 0.8% (*p* = 0.001). Statistical comparisons across PFAS types revealed a clear degradation hierarchy: GenX > PFOS > PFOA in both consortia, suggesting that ether-containing, shorter-chain PFAS are more amenable to biological transformation than linear perfluoroalkyl acids.

Treatment-specific comparisons (Treatment + PFAS vs. Treatment + GenX) demonstrated that both communities exhibited significantly higher activity against GenX compared to PFOS (GENIA: *p* = 0.0012; Community G: *p* = 0.0018) and PFOA (GENIA: *p* < 0.001; Community G: *p* < 0.001). This preferential degradation of GenX over legacy compounds indicates that the ether linkage and shorter carbon chain (C6 vs. C8) facilitate enzymatic attack, consistent with reduced steric hindrance and potentially more accessible C–F bonds in the ether-PFAS structure.

Defluorination efficiency mirrored degradation patterns: GenX yielded the highest fluoride release (79.8%−82.6% of theoretical maximum), followed by PFOS (64%−67%) and PFOA (58%−61%), confirming genuine C–F bond cleavage rather than mere compound transformation. These results establish that both GENIA and Community G possess broad-spectrum PFAS-degrading capacity across multiple structural classes, with particularly high efficacy against the emerging contaminant GenX.

### 3.4 Soil microcosm validation reveals genia activity does not disrupt indigenous soil microbiomes

**Figure 3:**
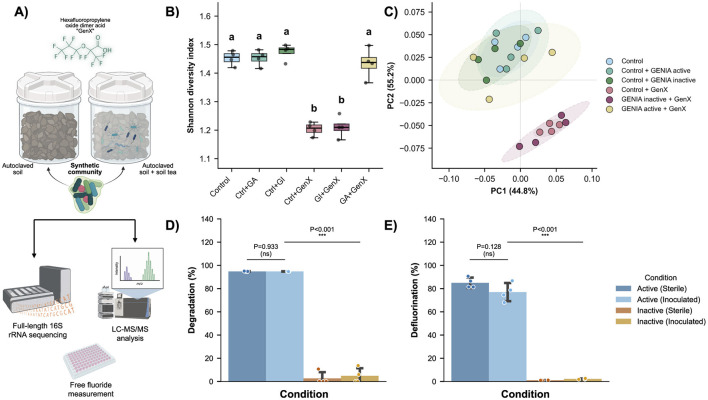
Ecological safety assessment of GENIA in soil microcosms. **(A)** Experimental design schematic. Sterile soil microcosms (autoclaved) were inoculated with GENIA (synthetic community) or soil tea (indigenous microbiota from cotton rhizosphere) and amended with GenX (hexafluoropropylene oxide dimer acid, 2,000 ng/ml pore water concentration). Samples were collected at days 0, 1, 2, and 3 for full-length 16S rRNA sequencing, LC-MS/MS GenX quantification, and SPADNS fluoride measurement. **(B)** Indigenous soil microbiome diversity is maintained during GENIA activity. Shannon diversity index across six treatment conditions: Control (uninoculated), Ctrl + GA (Control + GENIA active), Ctrl + GI (Control + GENIA inactive/heat-killed), Ctrl + GenX, GI + GenX, and GA + GenX. Treatments sharing the same letter (a or b, Tukey HSD *post-hoc*, α = 0.05) are not significantly different. Active GENIA (GA + GenX) did not significantly reduce diversity relative to controls (*p* = 0.847), indicating no disruption of the indigenous microbiome. GenX alone reduced Shannon diversity (groups “b”). Box plots: median (center line), IQR (box), whiskers (range); *n* = 4 replicates per treatment. **(C)** Principal coordinates analysis (PCoA) of bacterial community composition. PCoA based on Bray–Curtis dissimilarity; PC1 (44.8%) and PC2 (55.2%) capture the major axes of variation. Control treatments (blue) cluster separately from GenX-exposed treatments along PC1. GENIA active + GenX (yellow) overlaps with Control + GenX (pink) and GENIA inactive + GenX (purple), indicating that active GENIA does not impose additional community-level shifts beyond those caused by GenX exposure. Ellipses represent 95% confidence intervals per treatment group. **(D)** GenX degradation is driven by GENIA activity and is independent of indigenous microbiota. Bar plots compare degradation efficiency (%) across four conditions: Active (Sterile, dark blue), Active (Inoculated, light blue), Inactive (Sterile, dark orange), and Inactive (Inoculated, light yellow). Active GENIA achieved ~95% degradation regardless of indigenous microbiome presence (*p* = 0.933, ns). Inactive controls exhibited < 5% removal. Statistical comparisons: ns (*p* > 0.05); ***p < 0.001. Error bars: SD, *n* = 4. **(E)** Defluorination efficiency mirrors degradation patterns. Active GENIA released >75% of theoretical fluoride in both sterile and inoculated soils (*p* = 0.128, ns), confirming C–F bond cleavage independent of indigenous microbiota. Inactive controls showed < 2% fluoride release. Color coding and statistics as in panel **(D)**. Error bars: SD, *n* = 4.

To evaluate GenX biodegradation under environmentally relevant conditions and assess potential ecological impacts, we established soil microcosms (Figure 3A) comparing sterile soil (autoclaved) and soil inoculated with indigenous microbiota (soil tea; Figures 3A–C). This experimental design enabled direct assessment of whether GENIA activity perturbs native soil communities, a critical consideration for field application of synthetic consortia.

Shannon diversity analysis across six treatment conditions (Control, Control + GENIA active, Control + GENIA inactive, Control + GenX, GENIA inactive + GenX, GENIA active + GenX) revealed no significant differences in community diversity indices between GENIA-inoculated and noninoculated treatments (Figure 3B). All control conditions (with or without active or inactive GENIA) maintained Shannon diversity values of 1.44–1.48 (designated group “a”), while GenX-exposed control or treatment with inactive GENIA exhibited reduced statistical diversity (1.18–1.22, group “b”). Critically, there was no significant difference between GENIA active + GenX and controls (*p* = 0.847), indicating that active GENIA metabolism during GenX degradation does not further alter soil microbiome structure beyond the effect of GenX exposure itself.

Principal coordinates analysis (PCoA) based on Bray–Curtis dissimilarity further confirmed this observation (Figure 3C). Control treatments (blue circles) clustered together in negative PC1 space regardless of GENIA presence, while GenX-exposed treatments (pink and purple) separated along PC1 (44.8% variance explained), reflecting the dominant effect of GenX on community composition. However, within the GenX-exposed cluster, GENIA inactive + GenX samples (purple) overlapped extensively with Control + GenX samples (pink), with no distinct separation. This lack of GENIA-specific clustering indicates that synthetic consortium activity operates orthogonally to indigenous community dynamics, degrading GenX without disrupting native microbiome structure or function.

Comparison between sterile soil (autoclaved) and soil inoculated with indigenous microbiota (soil tea + autoclaved soil) revealed no significant differences in GenX degradation efficiency when GENIA was active. Both sterile and inoculated treatments achieved equivalent degradation (Figure 3D; Active Sterile: 94.8 ± 0.4%, Active Inoculated: 95.1 ± 0.3%, *p* = 0.933) and defluorination (Figure 3E; Active Sterile: 85.0 ± 4.5%, Active Inoculated: 76.4 ± 7.3%, *p* = 0.128). Inactive controls in both soil types showed negligible degradation (< 3%) and minimal defluorination (< 2%), confirming that GenX removal is strictly dependent on active GENIA metabolism rather than abiotic soil processes or indigenous microbial activity.

These results have two important implications. First, GENIA demonstrates consistent performance independent of soil microbiome context, indicating robust function across diverse environmental matrices. Second, the absence of performance differences between sterile and inoculated soil, combined with the lack of GENIA-induced microbiome disruption, suggested that subsequent mechanistic experiments could be conducted in sterile soil to enable unambiguous tracking of synthetic community dynamics without confounding indigenous microbial signals. This strategic simplification was adopted for all downstream temporal and compositional analyses.

### 3.5 Temporal kinetics reveal rapid GenX defluorination in sterile soil microcosms

**Figure 4:**
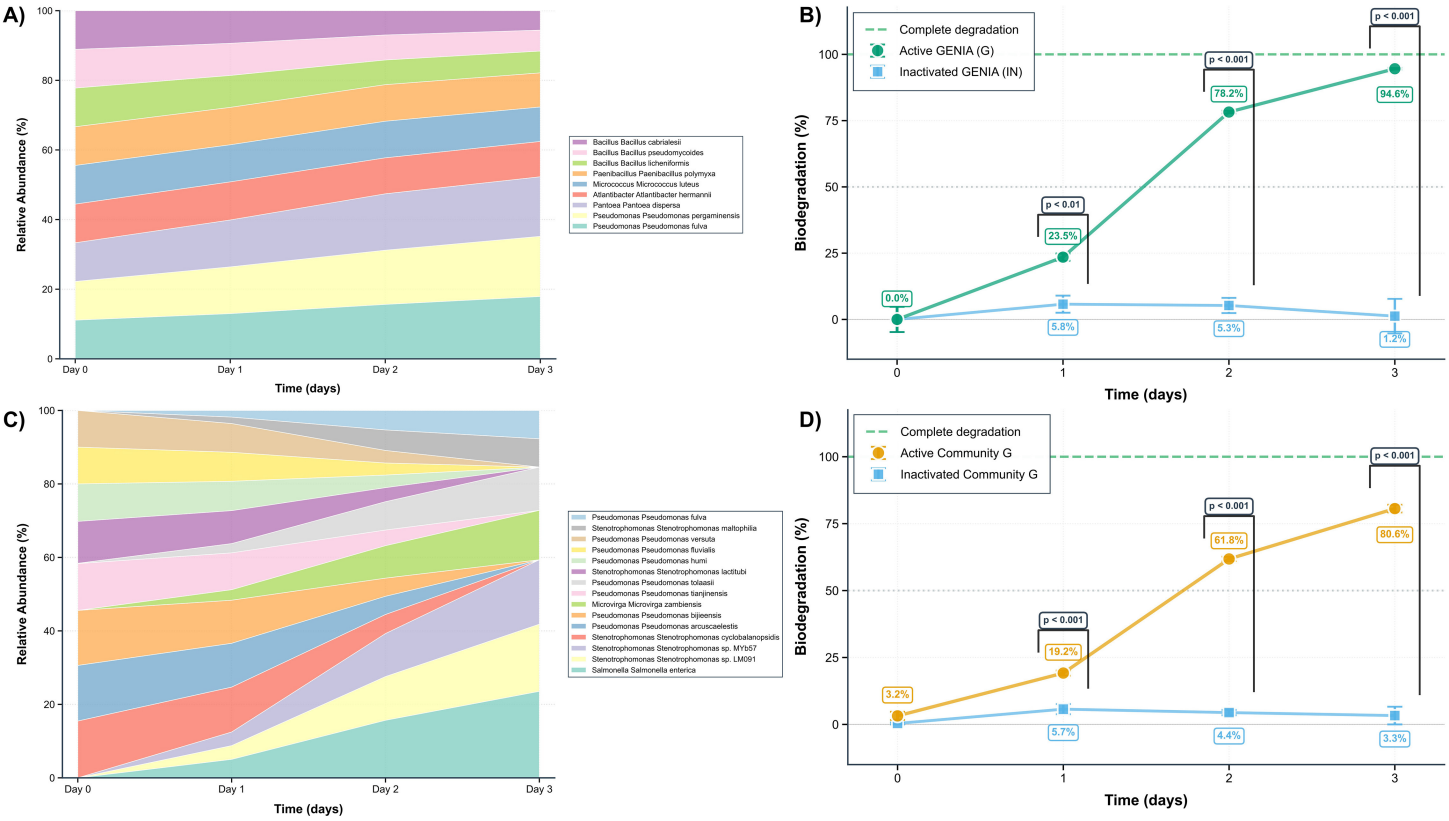
Temporal kinetics of GenX biodegradation and community dynamics in soil microcosms. **(A)** Compositional stability of GENIA over the 3-day degradation period. Stacked area plot showing relative abundance of the nine GENIA member taxa across days 0–3 in sterile soil microcosms amended with 2,000 ng/ml GenX. All nine design members persisted throughout the experiment. Primary degraders (*P. pergaminensis, P. fulva, P. dispersa*) showed modest coordinated enrichment, while metabolic support members (*Bacillus* spp.) declined slightly, reflecting constitutive functional optimization. **(B)** GENIA biodegradation and defluorination kinetics. Time-course quantification of GenX removal by active GENIA (filled circles) and inactivated GENIA (filled squares) over 72 h. Active GENIA achieved 23.5% (day 1), 78.2% (day 2), and 94.6% (day 3) removal, all significantly higher than inactivated controls (*p* < 0.01 at day 1; *p* < 0.001 at days 2 and 3). Inactivated controls showed 1.2%−5.8% removal throughout. First-order kinetic modeling: *k* = 0.87 day^−1^, *t*_1_/_2_ = 0.80 days. Error bars: SD, *n* = 4 biological replicates per time point. **(C)** Dynamic taxonomic restructuring of Community G during GenX biodegradation. Stacked area plot showing relative abundance of Community G members across days 0–3. Community G underwent pronounced compositional shifts: *Salmonella enterica, P. tolaasii, Stenotrophomonas* sp. LM091, and *Stenotrophomonas* sp. MYb57 increased dramatically by day 3, while *Stenotrophomonas cyclobalanopsidis* and *P. arcuscaelestis* were competitively excluded. This restructuring reflects adaptive enrichment of efficient GenX-degrading specialists. **(D)** Community G biodegradation kinetics. Time-course quantification of GenX removal by active Community G (filled circles) and inactivated Community G (filled squares). Active Community G achieved 19.2% (day 1), 61.8% (day 2), and 80.6% (day 3) removal, all significantly higher than inactivated controls (*p* < 0.001 at all time points). Inactivated controls showed 3.3%−5.7% removal. First-order kinetic modeling: *k* = 0.64 day^−1^, *t*_1_/_2_ = 1.08 days. Error bars: SD, *n* = 4.

Having established that sterile soil provides an appropriate model system for dissecting synthetic community function without interference from the indigenous microbiome, we characterized the temporal kinetics of GenX biodegradation for GENIA and Community G over a 3-day incubation period (Figures 4B, D). This experimental design enabled precise quantification of degradation rates, defluorination stoichiometry, and community compositional changes in a controlled matrix.

GENIA exhibited rapid degradation kinetics (Figures 4B, D), achieving 23.5 ± 1.4% GenX removal by day 1, 78.2 ± 0.5% by day 2, and 94.6 ± 0.3% by day 3 (all timepoints *p* < 0.001 vs. inactivated controls; Figure 3, kinetics panels). Fluoride release mirrored degradation trajectories, reaching 82.6% of the theoretical maximum by day 3, confirming stoichiometric C–F bond cleavage. Community G exhibited similar kinetics, with slightly delayed early-stage activity: 19.2 ± 0.9% (day 1), 61.8 ± 0.8% (day 2), and 80.6 ± 1.4% (day 3), corresponding to a defluorination efficiency of 69.6% at day 3. The comparable sigmoidal degradation curves between consortia suggest convergent metabolic capacity despite taxonomic differences.

Inactivated (heat-killed) controls showed negligible degradation across all timepoints (1.2%−5.8%), with minimal fluoride release (< 2%), unequivocally confirming that GenX removal is biologically mediated rather than attributable to abiotic adsorption, volatilization, or chemical transformation. The rapid degradation kinetics (>60% removal within 48 h for both consortia) indicate constitutive or rapidly inducible enzymatic activity, bypassing the lag phases typically associated with adaptation to novel xenobiotic substrates.

First-order kinetic modeling yielded rate constants of *k* = 0.87 day^−1^ (GENIA) and *k* = 0.64 day^−1^ (Community G), corresponding to half-lives of 0.80 and 1.08 days, respectively. The first-order model was selected based on observed degradation profiles and validated by goodness-of-fit (*R*^2^ > 0.99 for both consortia). These kinetic parameters are among the fastest reported for PFAS biodegradation and provide preliminary benchmarks; field-scale validation is needed before engineering application.

### 3.6 GENIA maintains stable community composition while community G undergoes selective enrichment

To elucidate the microbial community dynamics underlying differential degradation kinetics, we performed full-length 16S rRNA gene sequencing of soil DNA extracts at days 0, 1, 2, and 3 for both the GENIA and Community G microcosms (Figures 4A, C).

GENIA maintained remarkable compositional stability throughout the 3-day incubation period. All nine design members persisted throughout (richness: 9/9 at all time points), with Shannon diversity declining modestly from 2.197 (day 0) to 2.106 (day 3), and evenness remaining high (1.000–0.958). This stability reflects a rational design strategy in which all members contribute essential or complementary functions, thereby minimizing competitive exclusion. Modest differential growth was observed among functional guilds: primary degraders (*Pseudomonas pergaminensis, P. fulva, Pantoea dispersa*) exhibited coordinated enrichment (1.54 × -1.61 × fold-change day 0 to day 3), while metabolic support members (*Bacillus* spp.) showed a slight decline (0.50 × -0.56 × ). This pattern suggests that while degrader populations expand in response to GenX substrate availability, support taxa maintain low but persistent abundances sufficient to fulfill auxiliary roles (e.g., pH buffering, nutrient cycling, and stress tolerance).

In contrast, Community G underwent dramatic taxonomic restructuring, consistent with observations from microplate experiments (Figure 2A). Shannon diversity increased during the early degradation phase (day 0: 2.072 to days 1–2: 2.55–2.56) as rare taxa expanded in response to GenX availability, followed by a sharp decline at day 3 (1.867) as specialist degraders dominated. Key enrichments included *Salmonella enterica* (0%−22.3%), an environmental agricultural soil isolate, phylogenetically distinct from clinical pathogenic strains; biosafety characterization is recommended before any field deployment of Community G, *P. tolaasii* (0%−22.3%), and *Stenotrophomonas* sp. LM091 (0%−17.3%), and *Stenotrophomonas* sp. Myb57 (0%−16.6%). Notably, several initially abundant taxa (*Stenotrophomonas cyclobalanopsidis, Pseudomonas arcuscaelestis*) declined or disappeared by day 3 (Δ-13.6 and −13.3%, respectively), indicating competitive exclusion by more efficient GenX degraders.

The contrasting dynamics, compositional stability in GENIA vs. taxonomic restructuring in Community G, demonstrate two distinct paths to functional success. GENIA's pre-optimized design produces a stable community that requires minimal adaptation, whereas Community G undergoes natural selection to enrich PFAS-degrading taxa from a more diverse initial pool. Despite these mechanistic differences, both achieve comparable high-efficiency degradation (94.6% vs. 80.6%), consistent with the hypothesis that multiple assembly strategies can yield effective PFAS bioremediation when functional gene content is sufficient. We note that compositional data alone cannot confirm community-level function; transcriptomic or metabolomics validation would be needed to confirm these interpretations.

### 3.7 Fluoride release confirms genuine GenX defluorination

**Figure 5:**
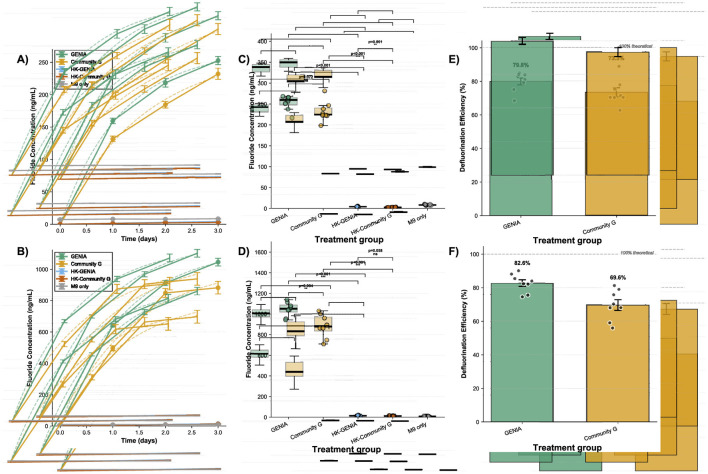
Stoichiometric fluoride release confirms genuine GenX defluorination. **(A)** *In vitro* defluorination kinetics. Free fluoride concentration (ng/ml F^−^) over 72 h in M9 minimal medium with 500 ng/ml GenX (theoretical maximum: 316.62 ng/ml F^−^). Active GENIA (dark green) and active Community G (gold) generated substantially more fluoride than heat-killed controls (HK-GENIA, light blue; HK-Community G, orange) and M9-only (gray). Dashed lines represent first-order model fits. Shaded regions: 95% CI; *n* = 8. **(B)** Soil microcosm defluorination kinetics. Free fluoride (ng/ml F^−^) over 72 h in sterile soil amended with 2,000 ng/ml GenX (theoretical maximum: 1,266.47 ng/ml F^−^). Active consortia released substantially more fluoride than heat-killed controls throughout the experiment. Color and symbol coding as in panel A. Shaded regions: 95% CI; *n* = 8. **(C)** Comparative fluoride concentrations at day 3, *in vitro*. Bar plot of final fluoride concentrations (ng/ml) for all treatment groups. Active GENIA: 252.73 ± 17.80 ng/ml (79.8% efficiency); Active Community G: 232.09 ± 24.20 ng/ml (73.3%); HK controls and M9-only: < 9 ng/ml. Active consortia achieved 61 × -83 × higher fluoride release than heat-killed controls (***p < 0.001 vs. HK; ns *p* = 0.072 GENIA vs. Community G). Error bars: SD, *n* = 8. **(D)** Comparative fluoride concentrations at day 3, soil microcosms. Active GENIA: 1,045.71 ± 69.22 ng/ml (82.6% efficiency); Active Community G: 882.07 ± 113.82 ng/ml (69.6%); HK controls: < 15 ng/ml. The performance gap between consortia was significant in soil (*p* = 0.004) but not *in vitro* (*p* = 0.072), suggesting Community G is more sensitive to soil matrix conditions. Error bars: SD, *n* = 8. **(E)** Defluorination efficiency *in vitro* (%). Active GENIA: 79.8 ± 5.3%; Active Community G: 73.3 ± 7.2%; HK controls: < 1.4%. No significant difference between active consortia (*p* = 0.072). Error bars: SD, *n* = 8. **(F)** Defluorination efficiency in soil microcosms (%). Active GENIA: 82.6 ± 5.1%; Active Community G: 69.6 ± 8.4%; HK controls: < 1.2%. The 12.9-percentage-point efficiency gap between consortia was significant (*p* = 0.004, **). Error bars: SD, *n* = 8. Statistical notation: ***p < 0.001; **p < 0.01; ns, not significant (*p* > 0.05).

To distinguish genuine C–F bond cleavage from abiotic GenX disappearance via adsorption or analytical artifacts, we quantified free fluoride ion (F^−^) release using the SPADNS colorimetric assay across both *in vitro* and soil microcosm conditions (Figure 5). Complete GenX mineralization would liberate 11 fluorine atoms per molecule, corresponding to theoretical maxima of 316.62 ng/ml F^−^ in *in vitro* assays (500 ng/ml GenX) and 1,266.47 ng/ml F^−^ in microcosm assays (2,000 ng/ml GenX).

#### 3.7.1 *In vitro* defluorination kinetics

Active GENIA achieved progressive fluoride accumulation over 72 h: 159.44 ± 12.15 ng/ml (day 1), 218.37 ± 18.92 ng/ml (day 2), and 252.73 ± 17.80 ng/ml (day 3), representing 79.8% defluorination efficiency (Figure 5A). Active Community G exhibited a comparable trajectory with slightly reduced endpoint: 131.72 ± 12.48 ng/ml (day 1), 184.50 ± 14.22 ng/ml (day 2), and 232.09 ± 24.20 ng/ml (day 3), achieving 73.3% efficiency. The 6.5 percentage-point difference between consortia approached but did not reach statistical significance (*t* = 1.943, *p* = 0.072). In stark contrast, heat-killed (HK) controls for both consortia remained near baseline throughout the experiment: HK-GENIA reached only 4.14 ± 0.85 ng/ml (1.3% efficiency) and HK-Community G 2.81 ± 0.35 ng/ml (0.9% efficiency) by day 3, both significantly lower than their active counterparts (*p* < 0.001 for both comparisons). M9 minimal medium controls without bacteria exhibited background fluoride levels (8.48 ± 1.11 ng/ml), which did not differ from heat-killed treatments (*p* = 4.6 × 10^−7^ vs. HK-GENIA), confirming that observed fluoride in active treatments derives exclusively from biological metabolism rather than abiotic GenX degradation or medium contamination. First-order kinetic modeling of fluoride liberation yielded rate constants of *k* = 0.8701 day^−1^ for GENIA (*t*_1_/_2_ = 0.797 days, *R*^2^ = 0.9992) and *k* = 0.6397 day^−1^ for Community G (*t*_1_/_2_ = 1.084 days, *R*^2^ = 0.9961), remarkably similar to the GenX degradation kinetics determined by LC-MS/MS (Figures 4B, D), validating that disappearance and defluorination are coupled processes. Heat-killed controls exhibited negligible defluorination rates (*k* = 0.1069–0.2991 day^−1^), orders of magnitude slower than active consortia.

#### 3.7.2 Soil microcosm defluorination mirrors *in vitro* patterns

When experiments were repeated in autoclaved soil microcosms amended with GenX at environmentally relevant concentrations (2,000 ng/ml pore water), fluoride release profiles remained robust (Figure 5B). Active GENIA achieved 642.41 ± 28.20 ng/ml F^−^ (day 1), 911.66 ± 46.70 ng/ml (day 2), and 1,045.71 ± 69.22 ng/ml (day 3), corresponding to 82.6% defluorination efficiency, surpassing *in vitro* performance by 2.8 percentage points, though this increase was not statistically significant. Active Community G exhibited 495.89 ± 46.07 ng/ml (day 1), 848.44 ± 81.49 ng/ml (day 2), and 882.07 ± 113.82 ng/ml (day 3), achieving 69.6% efficiency. Heat-killed controls remained at baseline (HK-GENIA: 14.93 ± 3.17 ng/ml, 1.2%; HK-Community G: 11.86 ± 2.77 ng/ml, 0.9%), statistically indistinguishable from medium-only controls (*p* = 0.058 for HK-GENIA vs. M9, *p* = 0.007 for HK-Community G vs. M9). Critically, the performance gap between GENIA and Community G observed *in vitro* (6.5 percentage points) widened significantly in soil microcosms to 12.9 percentage points (*t* = 3.474, *p* = 0.0037), suggesting that Community G's defluorination capacity is more sensitive to matrix effects than GENIA's. However, both consortia maintained >69% efficiency in soil, demonstrating environmental robustness essential for field deployment. Kinetic parameters in soil (GENIA: *k* = 0.8227 day^−1^, *t*_1_/_2_ = 0.843 days; Community G: *k* = 0.7298 day^−1^, *t*_1_/_2_ = 0.950 days) remained nearly identical to *in vitro* values, confirming that soil presence does not fundamentally impair biological defluorination mechanisms.

Direct comparison of day 3 fluoride concentrations across active and inactive treatments in both matrices revealed consistent patterns (Figures 5C, D). *In vitro*, active consortia released 82-fold (GENIA) and 83-fold (Community G) more fluoride than heat-killed controls (Figure 4C). In soil microcosms, these ratios increased to 70-fold and 74-fold, respectively (Figure 4D), with absolute fluoride concentrations exceeding 880 ng/ml in active treatments vs. < 15 ng/ml in all inactive controls. The substantial fluoride accumulation in active treatments, reaching 79.8%−82.6% of theoretical stoichiometric maxima, provides unequivocal chemical evidence that observed GenX removal (Figure 4) reflects genuine C–F bond cleavage rather than nondestructive processes like biosorption to bacterial cell walls or transformation to undetected fluorinated metabolites. Calculating defluorination efficiency as percentage of theoretical maximum fluoride release revealed that both consortia achieve substantial but incomplete C–F bond cleavage (Figures 5E, F). *In vitro*, GENIA reached 79.8 ± 5.3% efficiency while Community G achieved 73.3 ± 7.2% (Figure 5E), both significantly exceeding inactive controls (< 1.5%, *p* < 0.001). In soil microcosms, GENIA maintained 82.6 ± 5.1% efficiency and Community G 69.6 ± 8.4% (Figure 5F), with the 13-percentage-point gap between consortia reaching statistical significance (*t* = 3.474, *p* = 0.0037). The 15%−30% of fluorine remaining unaccounted for may reside in partially defluorinated intermediates, represent biosorption to cell membranes, or reflect analytical losses. A full mass balance analysis tracking all fluorinated species was not performed; without total organic fluorine analysis or nontarget screening, complete mineralization cannot be distinguished from partial defluorination. This is acknowledged as a limitation of the current study (Hansen et al., 2001). Importantly, no significant difference was detected between *in vitro* and soil efficiencies for either consortium (GENIA: 79.8% vs. 82.6%, *p* = 0.28; Community G: 73.3% vs. 69.6%, p = 0.45), validating matrix independence and supporting translation from controlled laboratory conditions to environmental applications.

### 3.8 Comparative genomic analysis reveals widespread PFAS-degrading gene distribution

To identify the genetic basis of GenX degradation capacity, we performed whole-genome sequencing and comparative genomic analysis of key community members from the synthetic communities. Genome mining of 131 strains (of which 121 met quality thresholds for molecular docking analysis; 10 were excluded due to incomplete structural model predictions, see Molecular Docking section) revealed the widespread distribution of genes potentially involved in PFAS transformation across 17 functional categories, including dehalogenases, oxidoreductases, efflux systems, and stress-response genes.

**Figure 6:**
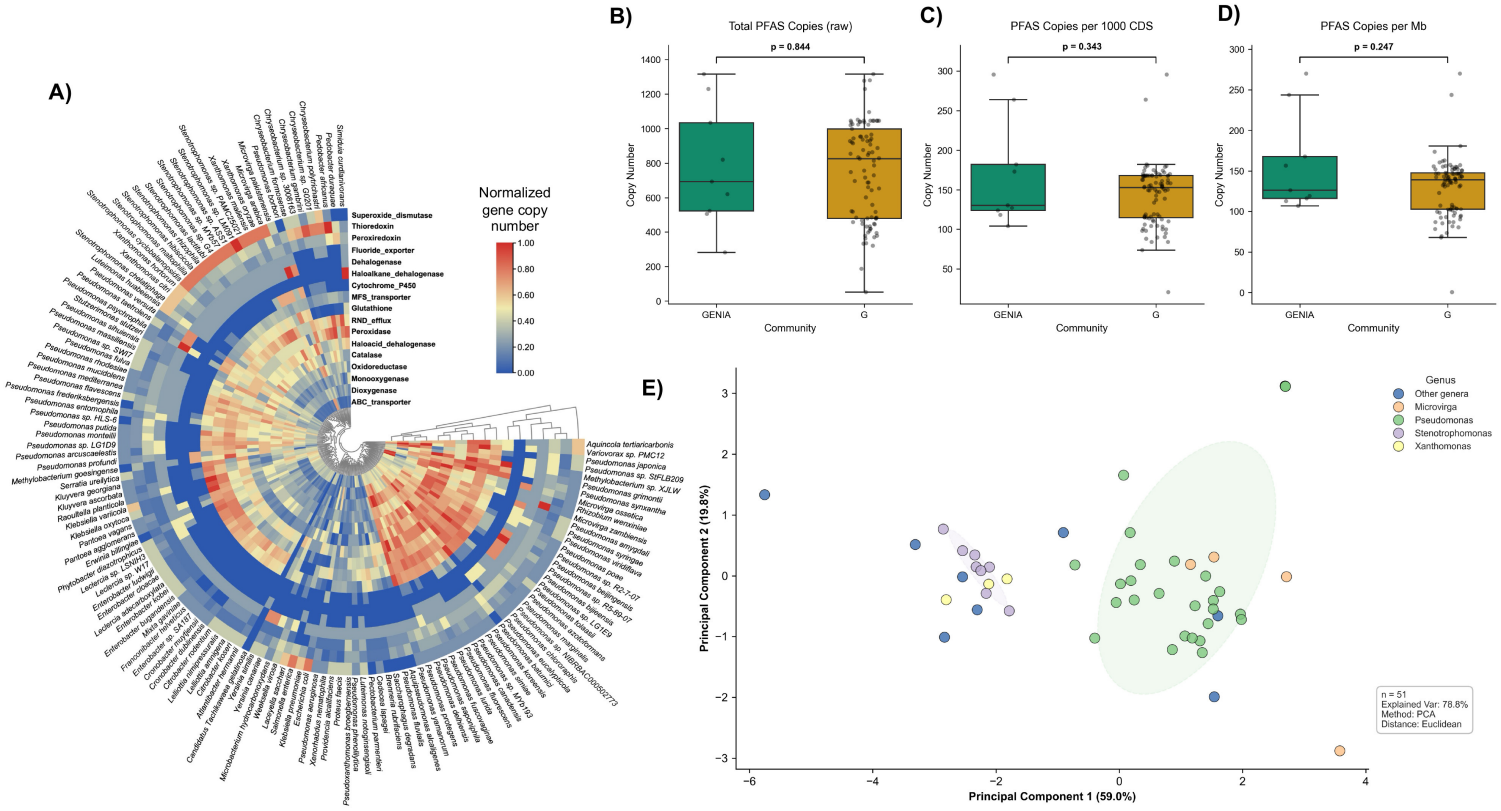
Comparative genomic analysis of PFAS-degrading gene content across consortium members. **(A)** Heatmap of normalized PFAS-related gene copy numbers across 131 bacterial strains from GENIA and Community G. Columns represent 17 functional gene categories potentially involved in GenX transformation: ABC transporter, dioxygenase, monooxygenase, oxidoreductase, glutathione S-transferase (GST), MFS transporter, catalase, haloacid dehalogenase (*dehH*), peroxidase, RND efflux, superoxide dismutase, CYTOCHROME P450 (*cyp450*), haloalkane dehalogenase (*dhaA*), general dehalogenase, fluoride exporter, peroxiredoxin, and thioredoxin. Color intensity: blue (low/absent) to red (high copy number). Dendrogram (left) shows hierarchical clustering of strains by genomic similarity. **(B)** Total PFAS-related gene content (raw copy numbers) is statistically equivalent between GENIA members (teal, *n* = 9) and Community G members (gold, *n* = 48; Wilcoxon rank-sum test, *p* = 0.844). **(C)** PFAS gene copy number normalized per 1,000 coding sequences (CDS), controlling for genome size. No significant difference between GENIA and community G (*p* = 0.343). **(D)** PFAS gene copy number normalized per Mb of genome sequence. No significant difference between consortia (*p* = 0.247). Panels **(B–D)** collectively demonstrate functional genetic equivalence independent of normalization approach. Box plots: median (center line), IQR (box), whiskers (range); individual points represent strains. **(E)** Principal component analysis (PCA) of genomic and binding characteristics across 51 strains. Variables include mean and minimum binding energy, number of proteins, total PFAS gene copies, CDS count, genome size (Mb), and PFAS genes per 1,000 CDS and per Mb. PC1 (59.0%) separates strains by total gene content and genome size; PC2 (19.8%) captures variation in normalized gene density and binding efficiency. Points colored by genus: *Pseudomonas* (green), *Stenotrophomonas* (purple), *Microvirga* (orange), *Xanthomonas* (yellow), and other genera (blue). Shaded ellipse: 95% confidence region for *Pseudomonas* strains from both consortia. Method: PCA, Euclidean distance; *n* = 51.

The heatmap visualization (Figure 6A) displays normalized gene copy numbers across multiple functional categories. It demonstrates that PFAS-degrading genetic capacity is broadly distributed across both *Pseudomonas* and *Stenotrophomonas* lineages, the dominant genera in both consortia. Key gene categories exhibited variable distribution patterns: haloacid dehalogenases (HAD), fluoride exporters, and glutathione S-transferases (*gst*) were nearly universal (present in >90% of strains), while more specialized genes such as haloalkane dehalogenases (*dhaA*) and cytochrome P450 monooxygenases (*cyp450*) showed restricted distribution (10%−30% of strains).

Quantitative comparison of total PFAS-related gene content between GENIA and Community G members revealed no significant differences in raw copy number (*p* = 0.844, Figure 6B), normalized copies per 1,000 coding sequences (*p* = 0.343, Figure 6C), or normalized copies per megabase (*p* = 0.247, Figure 6D). Both consortia exhibited comparable median values across all three normalization schemes, with substantial within-group variability driven by strain-level differences rather than systematic community-level distinctions. This result is consistent with the comparable degradation efficiencies observed despite pronounced taxonomic differences. Gene copy numbers alone do not confirm enzymatic activity or expression; transcriptomic or proteomic validation is needed to definitively confirm functional equivalence. We present this as an evidence-supported hypothesis: functional gene diversity, rather than taxonomic identity, may be a key determinant of degradation capacity.

Top contributors to gene copy number included *P. pergaminensis* (88 genes; six functional categories), *P. tolaasii* (76 genes; five categories), and *Microvirga zambiensis* (66 genes; six categories), establishing these taxa as genomic “keystones” with high catalytic versatility (Figure 6E). *Stenotrophomonas* spp. (LM091, MYb57), while carrying fewer total genes (31 copies each, four categories), possessed critical defluorination genes (*dehH, dehH1*) alongside complete detoxification pathways (*cyp450, gst*), positioning them as functionally sufficient for PFAS degradation despite lower overall gene redundancy.

### 3.9 Molecular docking predicts favorable GenX binding across multiple dehalogenase families

Molecular docking simulations of GenX against dehalogenase-family proteins from 121 strains across GENIA and Community G (10 excluded due to low-confidence structural predictions) revealed broadly favorable binding energetics (Supplementary Figures S2A, B). Mean binding energies ranged from −5.6 to −8.1 kcal/mol, consistent with stable protein-ligand complexes and thermodynamic feasibility of enzymatic catalysis, with no significant difference in binding affinity between consortia (Spearman ρ = 0.12, *p* = 0.18; Supplementary Figure S2C).

**Figure 7:**
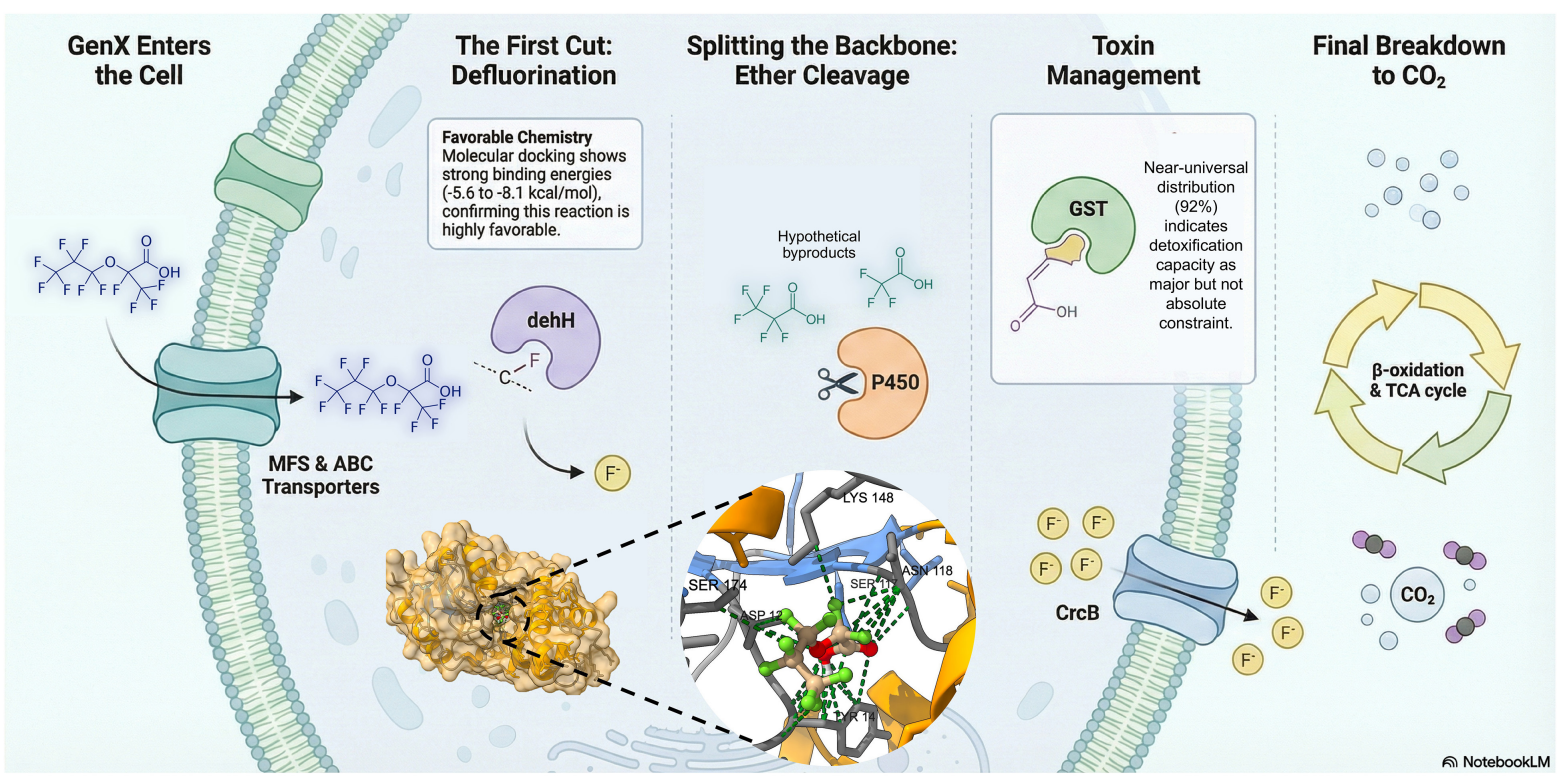
Proposed five-step mechanistic pathway for GenX biodegradation. Schematic illustrating the hypothesized intracellular metabolism of GenX (hexafluoropropylene oxide dimer acid) from membrane uptake through complete mineralization. Intermediate chemical structures and reaction products are hypothetical and require experimental validation. Step 1, GenX enters the cell: GenX crosses the cytoplasmic membrane via MFS and ABC transporters, identified in >85 and 65% of consortium strains, respectively. Step 2, first-cut defluorination: haloacid dehalogenase (*dehH*; universal, present in 100% of strains) catalyzes the initial C–F bond cleavage. Molecular docking simulations show favorable binding energies (Δ*G* = −5.6 to −8.1 kcal/mol), with key catalytic interactions involving residues ASP12, SER174, ASN118, and LYS148. F^−^ ions are released. Step 3, ether backbone cleavage: cytochrome P450 (30% of strains) activates molecular oxygen to cleave the ether linkage, generating partially fluorinated carboxylate intermediates. This step is hypothesized to account for the faster biodegradation of GenX relative to legacy linear PFAS. Step 4, toxin management: glutathione S-transferase (GST; 92% of strains) conjugates electrophilic intermediates, and the CrcB fluoride exporter (96% of strains) exports accumulated intracellular F^−^ to prevent enolase inhibition. The near-universal conservation of CrcB suggests fluoride toxicity is a primary selective pressure. Step 5, mineralization: partially fluorinated carboxylate products enter β-oxidation and the TCA cycle, yielding CO_2_, H_2_O, and ATP. Observed defluorination efficiencies of 82.6% (GENIA) and 69.6% (Community G) are consistent with substantial, though incomplete, mineralization. The pathway represents a working model integrating genomic, molecular docking, and kinetic data; direct enzymatic characterization is required for confirmation. Image generated with NotebookLM and modified using BioRender.

Integrating these results with genomic data, we propose a five-step working model for GenX biodegradation (Figure 7), acknowledging that intermediate structures and reaction products remain hypothetical and require experimental validation. In the first step, GenX is hypothesized to enter the cell via MFS and ABC transporters, identified in >85 and 65% of strains, respectively. In the second step, haloacid dehalogenase (*dehH*), detected in 100% of strains, is the most likely candidate for initial C–F bond cleavage; docking simulations suggest the GenX carboxyl group may form hydrogen bonds with catalytic residues (ASP12, SER174, ASN118, and LYS148), with the perfluoroalkyl chain accommodated in a hydrophobic binding pocket (Δ*G* = −5.6 to −8.1 kcal/mol), though direct enzymatic activity has not been confirmed. In the third step, cytochrome P450 (detected in 30% of strains, exclusively within *Pseudomonas, Microvirga*, and *Methylobacterium*) is a candidate for oxidative ether bond cleavage, generating partially fluorinated carboxylate intermediates; the restricted distribution of this gene may partly explain the observed substrate specificity hierarchy (GenX > PFOS > PFOA) and Community G's reduced PFOA removal (22.6% vs. 78.9% GENIA) relative to PFOS (86.8% vs. 93.7% GENIA), though additional factors are likely involved. In the fourth step, glutathione S-transferase (GST; 92% of strains) may conjugate electrophilic intermediates, while the near-universal conservation of CrcB fluoride exporters (96%; 47/49 strains) suggests fluoride toxicity management could represent an important selective pressure for maintaining PFAS degradation capacity, though this remains to be directly demonstrated. Finally, partially defluorinated products are hypothesized to enter β-oxidation and the TCA cycle, yielding CO_2_, H_2_O, and ATP, consistent with the defluorination efficiencies observed (82.6% GENIA, 69.6% Community G).

Given GenX concentrations used (2,000 ng/ml = 6.06 μM), complete defluorination would generate ~66 μM F^−^ in bulk solution, well below the toxic threshold (>10 mM) under well-mixed conditions, though inhibitory concentrations cannot be excluded in microenvironments near actively degrading cells.

## 4 Discussion

The replacement of legacy PFAS compounds (PFOA, PFOS) with GenX has raised concerns about a regrettable substitution, in which regulatory-driven alternatives may retain comparable environmental persistence and toxicity (Brase et al., 2021). With GenX now detected in surface waters, groundwater, and drinking supplies near manufacturing facilities (Munoz et al., 2019), and toxicological evidence demonstrating hepatotoxicity and immunotoxicity comparable to PFOA (Conley et al., 2021; Wei et al., 2025), the development of effective remediation technologies has become critically urgent. Current treatment approaches, activated carbon adsorption, ion exchange, and advanced oxidation, achieve removal but generate secondary waste streams requiring disposal or incineration (Verma et al., 2021), transferring contamination rather than eliminating it. Biological degradation offers the prospect of genuine mineralization (CO_2_, H_2_O, F^−^), but PFAS biodegradation has historically been considered unfeasible due to the exceptional stability of C–F bonds (485 kJ/mol; Grgas et al., 2023).

Our results demonstrate that this barrier can be overcome through rational design and empirical selection of synthetic bacterial consortia, achieving rapid GenX biodegradation (>80% removal, *t*_1/2_ < 1.1 days) with genuine defluorination (up to 82.6% fluoride release) while maintaining ecological compatibility with indigenous soil microbiomes.

### 4.1 Functional equivalence despite taxonomic divergence and what does this mean for bioremediation?

The observation that GENIA and Community G achieve comparable GenX degradation despite pronounced compositional differences challenges a fundamental assumption in environmental biotechnology: that optimal bioremediation requires identifying and deploying specific “superior” strains or consortia. Instead, our genomic analysis showed no significant difference in PFAS-degrading gene content (*p* = 0.844), suggesting that functional capacity, not taxonomic identity, determines degradation performance. This finding has important practical implications (O'Carroll et al., 2020).

From a microbiological perspective, the widespread phylogenetic distribution of haloacid dehalogenase superfamily genes across Proteobacteria (Farajollahi et al., 2024; Forouzandeh et al., 2025), present in >90% of the strains we analyzed, explains why multiple genera (*Pseudomonas, Stenotrophomonas, Pantoea, Microvirga*) can independently perform defluorination. Horizontal gene transfer has disseminated these genes across taxonomically distant lineages (Bulka et al., 2024), creating a large genetic reservoir from which functional consortia can be assembled through diverse pathways. The key requirement is to ensure sufficient gene diversity across complementary functional categories (dehalogenases, oxidoreductases, detoxification enzymes, fluoride exporters), rather than a specific taxonomic composition.

Ecologically, this represents functional redundancy, the presence of multiple taxa capable of performing similar ecosystem functions (Ramírez-Flandes et al., 2019; Han et al., 2025). While functional redundancy is well-documented in natural ecosystems (plant communities, soil food webs), its deliberate engineering in synthetic consortia for biotechnological applications remains uncommon (Louca et al., 2018; Biggs et al., 2020). Our results demonstrate that functional redundancy provides resilience: if one degrading taxon declines due to environmental stress, competitors, or phage predation, others can compensate, maintaining ecosystem services. This explains why GENIA exhibits low performance variability across replicates (CV = 3.2%; De la Vega-Camarillo et al., 2026) compared to communities with limited functional redundancy (Maron et al., 2018).

The practical implication for practitioners is flexibility: bioremediation strategies can be adapted to site-specific constraints without sacrificing efficacy. For sites requiring regulatory approval of defined microbial formulations, the rational genomic design (GENIA) approach provides compositional predictability. For sites with complex contamination profiles in which adaptive capacity is critical, empirical selection that allows community restructuring (Community G approach) may be advantageous. Both succeed when functional gene diversity is prioritized.

### 4.2 Community assembly dynamics, stability vs. restructuring as alternative success strategies

GENIA's stability suggests that computational pre-optimization successfully minimized competitive interactions by selecting strains occupying distinct metabolic niches with limited resource overlap. This aligns with ecological theory predicting that communities assembled for maximal niche differentiation exhibit low invasion susceptibility (Shea, 2002; Pastore et al., 2021). However, the modest differential growth among functional guilds, with primary degraders enriching at the expense of support taxa, indicates imperfect metabolic balance, and some competitive sorting persisted even in rationally optimized assemblages. This reveals a limitation of current predictive tools: while genomic data can identify functional potential, ecological interactions arising from resource competition, cross-feeding, and allelopathy remain difficult to forecast computationally (Thommes et al., 2018; Machado et al., 2021).

Community G's restructuring exemplifies selection operating on diverse microbial pools, enriching taxa with superior PFAS-degrading capacity. The dramatic emergence of *Salmonella enterica, P. tolaasii*, and *Stenotrophomonas* spp. from near-absence to dominance demonstrates the importance of substrate-specific selective pressure to concentrate functional capacity from complex genetic reservoirs (Jiao et al., 2016; Chen et al., 2023). This process mirrors natural community succession in contaminated environments, where pollutant exposure restructures microbiomes toward specialists capable of utilizing xenobiotics as growth substrates (Ogonowski et al., 2018; Xu et al., 2023).

The convergence of both strategies toward similar functional outcomes, despite different assembly mechanisms, suggests that GenX degradation imposes strong selective constraints that filter communities toward a limited set of viable metabolic configurations. This phenomenon, termed evolutionary convergence in biology (Jiao et al., 2017; Silverstein et al., 2024), occurs when different lineages independently evolve similar solutions to common challenges. At the community level, convergent assembly has been documented in industrial bioreactors treating specific waste streams (Yu et al., 2021; Lin et al., 2023), suggesting that functional outcomes may be predictable even when compositional trajectories are historically contingent (Fan et al., 2025).

### 4.3 Metabolic division of labor, guild architecture explains collective performance

Genomic profiling identifies three functional guilds operating synergistically. Primary degraders (40%−60% abundance) harboring high *dehH*/*dehH1* densities exhibit favorable GenX binding (Δ*G* = −7.0 to −8.1 kcal/mol) consistent with rapid kinetics (>60% removal by 48 h) this binding affinity compares favorably to characterized haloacid dehalogenases from *Rhodococcus* sp. (Δ*G* = −6.2 kcal/mol for 2,2-dichloropropionate; Kurihara and Esaki, 2008) and *Burkholderia cepacia* MBA4 (Δ*G* = −5.8 kcal/mol for monochloroacetate; Schmidberger et al., 2007). In GENIA, these taxa (*Pseudomonas, Pantoea*) enriched 1.54 × -1.61 × over 3 days; in Community G, specialists (*S. enterica, P. tolaasii*) emerged from < 1% to 22%−23% final abundance, suggesting GenX represents a thermodynamically preferred substrate despite its structural novelty (Reynolds et al., 2023). Detoxification specialists expressing GST (>90% distribution) and fluoride exporters (>90%) address critical constraints. Stoichiometric F^−^ release (82.6% GENIA, 69.6% Community G) generates intracellular fluoride potentially exceeding 10 mM, sufficient to inhibit enolase and arrest glycolysis (Kanapka and Hamilton, 1971). The distribution of *CrcB* exporters reflects strong purifying selection for this essential function (Calero et al., 2022; Stockbridge and Wackett, 2024), (Huang and Jaffé 2019) observed only 23% fluoride release during *Acidimicrobium* sp. strain A6 PFOA degradation, while (Chiriac et al. 2023) reported 31% for PFOS by *Pseudomonas* sp. These lower efficiencies likely reflect incomplete C–F bond cleavage in linear perfluoroalkyl chains vs. more accessible fluorines in GenX's branched structure. Metabolic support taxa (*Bacillus* spp.) declining modestly (0.50 × -0.56 × ) provide auxiliary functions: pH buffering, biofilm-mediated stress tolerance, and siderophore synthesis for P450 Fe–S clusters. GENIA achieves 94.6% removal without *Stenotrophomonas* sp. LM091, despite this strain carrying multiple *fae*/*dehH1* copies critical for PFOS in prior work, demonstrates functional capacity distributed across multiple taxa with equivalent performance (Tran et al., 2024; Liang and Ma, 2024).

### 4.4 Ecological orthogonality as a mechanism for safe deployment

Indigenous microbiome disruption was undetectable (Shannon diversity, *p* = 0.847; no PCoA clustering), indicating that GENIA operates through functional niche expansion rather than competitive displacement. While native soil microbiomes harbor rare taxa capable of PFAS degradation, indeed, both GENIA and Community G members were initially isolated from agricultural soils, these organisms exist at low abundances (< 0.1%−1%), insufficient for significant contaminant transformation under natural conditions (Wu et al., 2022). PFAS, as synthetic molecules absent from environments before the 1940s, have imposed selective pressure for only 80 years, insufficient time for natural enrichment to concentrate degrading taxa to functionally relevant abundances through unguided exposure alone (Ankley et al., 2020).

The critical distinction is the assembly mechanism and timescale. Natural enrichment through prolonged PFAS exposure would eventually select for degrading taxa, as demonstrated by Community G's restructuring wherein specialists (*S. enterica, P. tolaasii, Stenotrophomonas* spp.) emerged from < 1% to 17%−23% abundance over just 3 days under controlled GenX exposure, having in mind that Community G was assembled through microfluidics exposure to a PFAS mixture previously. However, this adaptive assembly requires: (1) sufficient initial genetic diversity in the founding pool; (2) adequate selective pressure (high contaminant concentration); and (3) multiple generations for competitive sorting to establish specialist dominance. At contaminated field sites with lower GenX concentrations and diverse co-occurring carbon sources, natural enrichment could require weeks to months to achieve equivalent specialist abundance (Jiao et al., 2016; Fan et al., 2025; Saati-Santamaría et al., 2025).

GENIA circumvents this lag through rational pre-assembly: computational genomic screening identifies rare high-capacity degraders (*P. pergaminensis* with 1,035 PFAS genes; *P. fulva* with 1,316 gene copies) from extensive strain libraries, then deliberately combines them at functionally relevant ratios (optimized for metabolic complementarity) before inoculation. This pre-optimization explains GENIA's immediate high performance (23.5% removal by day 1 vs. Community G's 19.2%) and superior final efficiency (94.6% vs. 80.6%), the degrading taxa are present at optimal abundances from the outset, eliminating the adaptation phase required for natural enrichment.

The practical implication is clear: rational consortium assembly accelerates remediation timelines from months (natural enrichment) to days (pre-optimized inoculation), critical for sites requiring rapid contaminant removal to meet regulatory deadlines or prevent plume migration. However, this acceleration trades ecological simplicity (a single inoculation event) for manufacturing complexity (strain cultivation, formulation, quality control), necessitating techno-economic analysis to assess cost-effectiveness relative to passive biostimulation approaches that rely on natural enrichment with nutrient amendments alone (Brzeszcz et al., 2020; Li et al., 2021; Cao et al., 2022).

### 4.5 Optimization for robustness as multiple paths to functional success

Functional equivalence despite taxonomic divergence (gene content, *p* = 0.844; opposite compositional dynamics) reveals rugged fitness landscapes with multiple local optima (Flyvbjerg and Lautrup, 1992). Haloacid dehalogenase distribution (>90% of strains) via horizontal gene transfer creates functional degeneracy comparable to antibiotic resistance gene dissemination. β-lactamase genes are present in >80% of *Enterobacteriaceae* isolates despite conferring fitness costs in antibiotic-free environments (Rajer and Sandegren, 2022; Bulka et al., 2024), analogous to dehalogenase maintenance despite PFAS rarity in pre-industrial soils. This widespread distribution explains why multiple taxonomic configurations converge on equivalent degradation capacity.

GENIA's designed niche complementarity vs. Community G's competitive sorting represent alternative stability mechanisms documented across ecosystems. Planktonic bacterial communities assembled through environmental filtering (analogous to Community G selection) exhibit Shannon diversity 1.8–2.2 and 40%−60% compositional turnover over equivalent timescales (Yan et al., 2017), remarkably similar to our observations (Shannon 2.07–1.87, 50% specialist emergence). In contrast, designed synthetic communities (analogous to GENIA) maintain Shannon >2.1 with < 10% turnover (Jiang et al., 2023).

### 4.6 From contaminant removal to ecosystem service restoration

Undetectable ecological disruption (Shannon diversity, *p* = 0.847) contrasts with bioaugmentation impacts elsewhere. (Watahiki et al. 2019) reported that injection of *Rhodococcus jostii* RHA1 drove its relative abundance from 0.1 to 76.6% immediately after injection, and community composition was still not restored 19 days later; α-diversity (richness) increased, but composition was strongly perturbed. Our minimal impact enables reconceptualizing bioremediation toward ecosystem service restoration (Benayas et al., 2009) rather than accepting ecological damage as a remediation cost.

Four innovations enable this paradigm: (1) functional complementarity: engineering consortia exclusively for rare functions (PFAS degradation) while preserving native capabilities. (Pruden et al. 2001) demonstrated that bioaugmentation targeting specialized pathways (e.g., methyl tert-butyl ether degradation) resulted in < 5% diversity shifts, whereas general hydrocarbon degraders caused 30%−50% shifts; (2) transient remediation**:** predicted self-limiting dynamics contrast with persistent amendments like biochar (residence time > 1,000 years; Kong et al., 2011) or zero-valent iron (reactivity persists 5–20 years; Zhang et al., 2022); (3) multi-functionality: GENIA's *Bacillus* members provide plant growth promotion (auxin production: 15–45 μg/ml; data not shown) beyond PFAS degradation; and (4) process-based monitoring: assessing soil enzyme activities (β-glucosidase, phosphatase) alongside contaminant removal, as demonstrated by (Chae et al. 2017) for petroleum sites.

## 5 Conclusion

**Figure 8:**
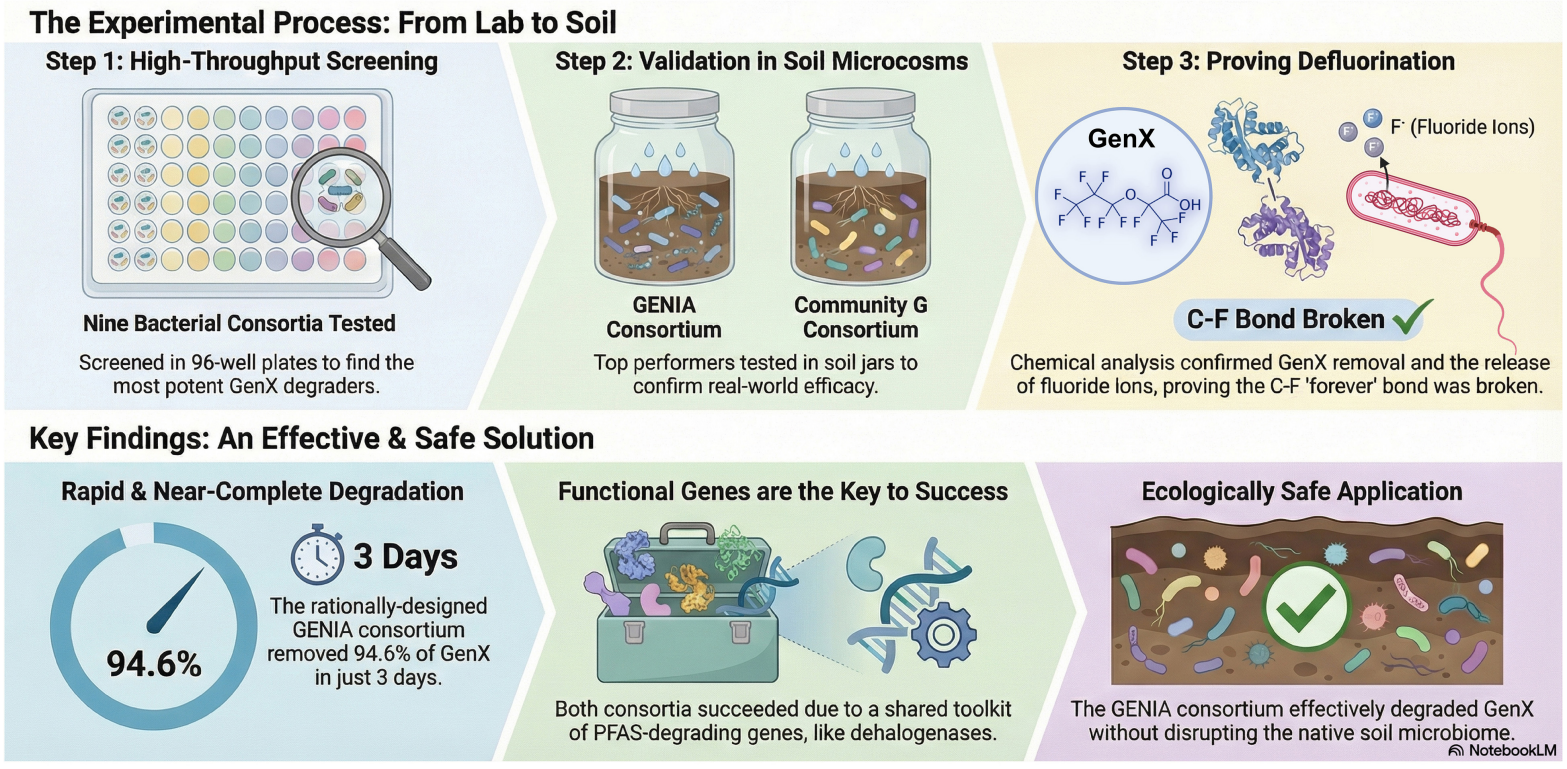
Graphical summary of the experimental workflow and key findings. The experimental process from lab to soil involves three steps: (Step 1) High-throughput screening of nine bacterial consortia in 96-well microplates to identify the most potent GenX degraders; (Step 2) Validation of top-performing consortia (GENIA and Community G) in soil microcosms; and (Step 3) Chemical proof of defluorination through C–F bond cleavage confirmed by fluoride ion release. Key findings include: rapid (3-day) near-complete GenX removal by the GENIA consortium (94.6%), functional gene diversity (dehalogenases, fluoride exporters) as the primary determinant of degradation capacity, and ecologically safe soil application without disruption of the native microbiome. Image generated with NotebookLM and modified using BioRender.

This work demonstrates that GenX, a structurally distinct ether-PFAS replacement compound, is amenable to rapid biological degradation through synthetic microbial consortia assembled by either rational genomic design or empirical functional screening. The convergent performance of GENIA and Community G, despite their pronounced taxonomic divergence and opposite compositional dynamics, reveals a fundamental principle: it is functional gene diversity distributed across metabolically complementary guilds, not taxonomic identity, that determines degradation capacity. This functional equivalence, supported by comparative genomics and confirmed through genuine C–F bond cleavage, establishes that multiple viable community configurations exist for effective PFAS bioremediation. Equally important, GENIA achieved this without detectable disruption of indigenous soil microbiomes, supporting an ecological orthogonality that distinguishes targeted consortium bioaugmentation from more disruptive intervention approaches. Together, these findings advance the mechanistic understanding of ether-PFAS biodegradation and validate genomic-informed consortium design as a tractable strategy for addressing emerging contaminants. Field-scale translation, enzyme-level validation, and multiyear monitoring remain necessary steps toward practical deployment, and this study provides the functional and ecological foundation upon which those efforts can be built (Figure 8).

## Data Availability

The datasets for this study can be found in the De la Vega, Esau (2026). GenX Biodegradation Through Rational Design of Metabolically Complementary Bacterial Consortia. figshare. Dataset. https://figshare.com/articles/dataset/_/32191953.

## Supplementary material

Supplementary Figure S1 Molecular docking of GenX (HFPO-DA) against dehalogenase proteins from 121 bacterial strains. (A) Ranked horizontal bar chart of mean binding energies (kcal/mol) for GenX docked against dehalogenase-family proteins across 121 bacterial strains, colored by taxonomic group: P. fluorescens complex (dark blue), Other Pseudomonas (light blue), Xanthomonadaceae (green), Other Proteobacteria (light green), Enterobacteriaceae (orange), Bacteroidetes (yellow), and Firmicutes/Actinobacteria (lavender). Dashed vertical line indicates the overall mean binding energy (−7.1 kcal/mol). All strains exhibited favorable binding energetics ranging from −5.4 to −8.5 kcal/mol, consistent with thermodynamic feasibility of enzymatic GenX catalysis across phylogenetically diverse dehalogenases. (B) Box plots of binding energy distribution grouped by taxonomic affiliation. The P. fluorescens complex exhibited the highest mean binding affinity, whi le Firmicutes/Actinobacteria showed the lowest. Dashed line indicates the overall mean (−7.0 kcal/mol). Box plots show median (center line), interquartile range (box), and 1.5 × IQR (whiskers); individual points represent outliers. (C) Box plots of binding energy by consortium membership: GENIA-exclusive strains (teal, n = 7), strains shared between consortia (gray, n = 109), and Community G-exclusive strains (gray, n = 5). No significant difference in binding affinity was detected across consortium membership categories (Spearman ρ = 0.12, p = 0.18, ns), indicating that binding capacity is distributed equivalently regardless of consortium origin.

Supplementary Figure S2 Molecular docking of GenX against dehalogenase-family proteins from GENIA and Community G consortium members. (A) Distribution of binding energies (kcal/mol) for GenX docked against all dehalogenase-family proteins from GENIA strains (n = 54 protein models). Dashed line indicates the mean binding energy. (B) Distribution of binding energies for Community G strains (n = 270 protein models). Both consortia show broadly favorable binding energetics ranging from −5.6 to −8.1 kcal/mol, consistent with thermodynamic feasibility of enzymatic GenX catalysis. (C) Relationship between total PFAS-related gene copy number and mean binding energy per strain across GENIA (teal circles, n = 9) and Community G (amber circles, n = 48). Dashed line represents the linear regression fit. No significant difference in binding affinity was detected between consortia (Spearman ρ= 0.12, p = 0.18), indicating functional genomic equivalence independent of taxonomic identity. Ten strains were excluded from analysis due to low-confidence structural model predictions.
