# Supplementary figures and images for: Convergent GenX biodegradation by genomically designed and functionally screened synthetic bacterial consortia
Source: Front Microbiol. 2026 Jun 24;17:1848796. doi: 10.3389/fmicb.2026.1848796 (PMC13344493; doi:10.3389/fmicb.2026.1848796)

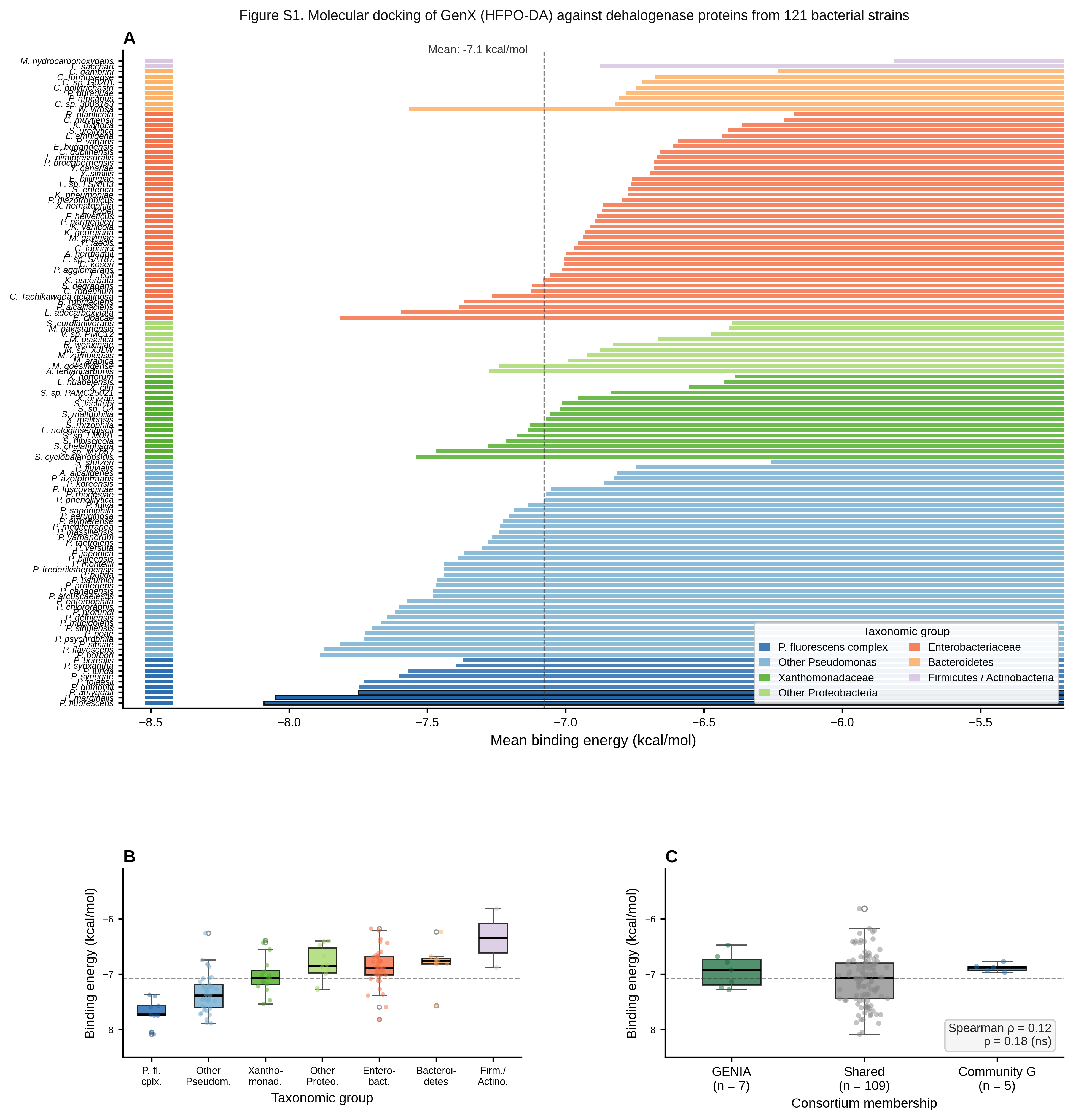

Supplement: Supplementary Figure S1 — Molecular docking of GenX (HFPO-DA) against dehalogenase proteins from 121 bacterial strains. (A) Ranked horizontal bar chart of mean binding energies (kcal/mol) for GenX docked against dehalogenase-family proteins across 121 bacterial strains, colored by taxonomic group: P. fluorescens complex (dark blue), Other Pseudomonas (light blue), Xanthomonadaceae (green), Other Proteobacteria (light green), Enterobacteriaceae (orange), Bacteroidetes (yellow), and Firmicutes/Actinobacteria (lavender). Dashed vertical line indicates the overall mean binding energy (−7.1 kcal/mol). All strains exhibited favorable binding energetics ranging from −5.4 to −8.5 kcal/mol, consistent with thermodynamic feasibility of enzymatic GenX catalysis across phylogenetically diverse dehalogenases. (B) Box plots of binding energy distribution grouped by taxonomic affiliation. The P. fluorescens complex exhibited the highest mean binding affinity, whi le Firmicutes/Actinobacteria showed the lowest. Dashed line indicates the overall mean (−7.0 kcal/mol). Box plots show median (center line), interquartile range (box), and 1.5 × IQR (whiskers); individual points represent outliers. (C) Box plots of binding energy by consortium membership: GENIA-exclusive strains (teal, n = 7), strains shared between consortia (gray, n = 109), and Community G-exclusive strains (gray, n = 5). No significant difference in binding affinity was detected across consortium membership categories (Spearman ρ = 0.12, p = 0.18, ns), indicating that binding capacity is distributed equivalently regardless of consortium origin. [file Image_1.png]

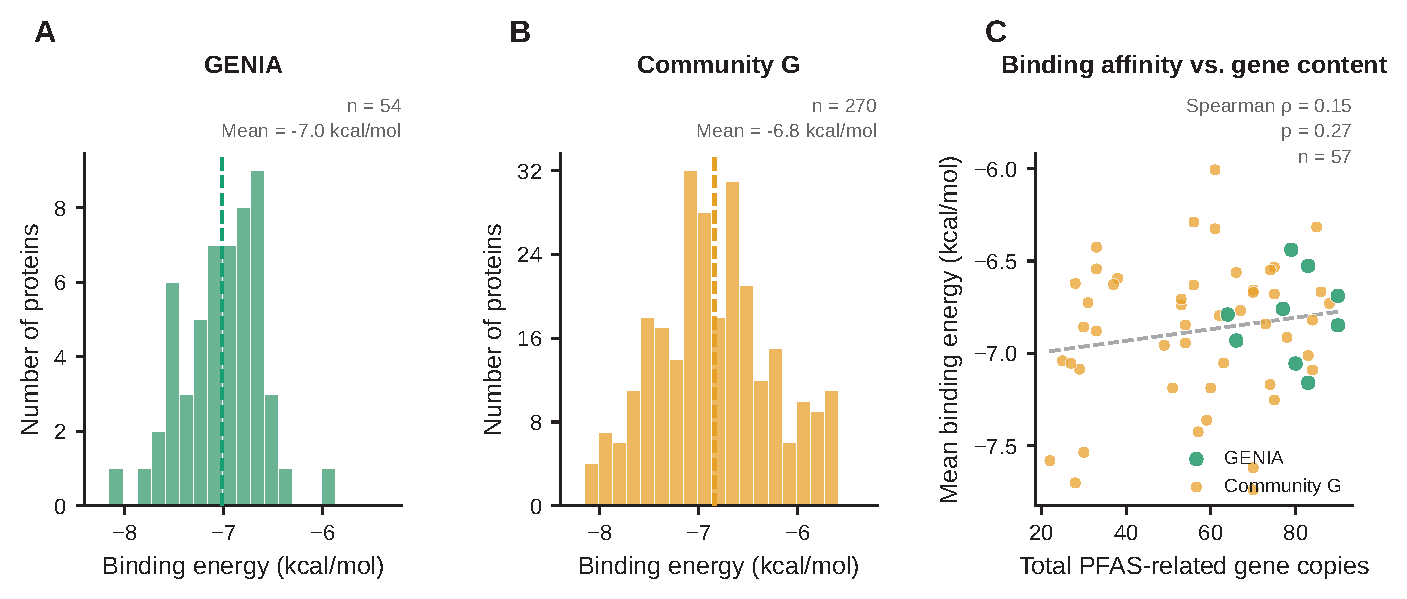

Supplement: Supplementary Figure S2 — Molecular docking of GenX against dehalogenase-family proteins from GENIA and Community G consortium members. (A) Distribution of binding energies (kcal/mol) for GenX docked against all dehalogenase-family proteins from GENIA strains (n = 54 protein models). Dashed line indicates the mean binding energy. (B) Distribution of binding energies for Community G strains (n = 270 protein models). Both consortia show broadly favorable binding energetics ranging from −5.6 to −8.1 kcal/mol, consistent with thermodynamic feasibility of enzymatic GenX catalysis. (C) Relationship between total PFAS-related gene copy number and mean binding energy per strain across GENIA (teal circles, n = 9) and Community G (amber circles, n = 48). Dashed line represents the linear regression fit. No significant difference in binding affinity was detected between consortia (Spearman ρ= 0.12, p = 0.18), indicating functional genomic equivalence independent of taxonomic identity. Ten strains were excluded from analysis due to low-confidence structural model predictions. [file Image_2.png]
